# Seneca Valley virus induces mitochondrial apoptosis by activating ER stress or the PERK pathway based on Ca^2+^ transfer from ER to mitochondria

**DOI:** 10.1128/jvi.02177-24

**Published:** 2025-02-06

**Authors:** Lei Hou, Xiaoyu Yang, Changzhe Liu, Ju Yu, Zhi Wu, Yong Wang, Penghui Zeng, Jinshuo Guo, Yongyan Shi, Jianwei Zhou, Jue Liu

**Affiliations:** 1 College of Veterinary Medicine, Yangzhou University 614704 , Yangzhou, China; 2 Jiangsu Co-Innovation Center for Prevention and Control of Important Animal Infectious Diseases and Zoonoses, Yangzhou University 38043 , Yangzhou, China; 3 College of Animal Science and Technology, Anhui Agricultural University 605541 , Hefei, China; University of Kentucky College of Medicine, Lexington, Kentucky, USA

**Keywords:** Seneca Valley virus, ER stress, Ca^2+^, mitochondrial dysfunction, apoptosis

## Abstract

Seneca Valley virus (SVV), also known as Senecavirus A, a porcine pathogen that causes vesicular diseases, is prevalent in pig herds worldwide. SVV infection induces endoplasmic reticulum (ER) stress in PK-15 and BHK-21 cells, accompanied by activation of the protein kinase R (PKR)-like endoplasmic reticulum kinase (PERK) and activating transcription factor 6 (ATF6) pathways, which in turn facilitates SVV replication. ER stress is associated with the regulation of Ca^2+^ homeostasis and mitochondrial apoptosis. However, the precise role of Ca^2+^ in SVV-induced apoptosis remains unclear. In this study, western blotting, flow cytometry, and terminal deoxynucleotidyl transferase-mediated dUTP-biotin nick-end labeling (TUNEL) detection revealed that either ER stress or the PERK pathway is involved in the apoptosis of SVV-infected cells treated with specific inhibitors. Furthermore, SVV-mediated ER stress markedly contributed to the transfer of Ca^2+^ from the ER to mitochondria. The subsequent increase in mitochondrial Ca^2+^ content was accompanied by an increased number of ER membranes near the mitochondria. Finally, the inhibition of mitochondrial Ca^2+^ overload, ER stress, and the PERK pathway substantially attenuated SVV-mediated mitochondrial dysfunction, as evidenced by analyzing mitochondrial membrane potential (MMP), mitochondrial permeability transition poremPTP, reactive oxygen speciesROS, and adenosine 5′-triphosphate ATP, and the levels of mitochondrial apoptosis. These findings demonstrate that SVV induces mitochondrial apoptosis, which is dependent on ER stress-mediated transmission of Ca^2+^ from the ER to the mitochondria.

**IMPORTANCE:**

Viruses have developed multiple mechanisms to facilitate their proliferation or persistence through manipulating various organelles in cells. Seneca Valley virus (SVV), as a novel emerging pathogen associated with vesicular disease, is clinically and economically important infections that affect farm animals. Previously, we had confirmed that SVV-induced endoplasmic reticulum (ER) stress benefited for viral replication. Ca^2+^, as an intracellular signaling messenger mainly stored in the ER, is regulated by ER stress and then involved in apoptosis. However, the precise mechanism that Ca^2+^ transfer induced by SVV infection triggered apoptosis remained unclear. Here, we found that SVV infection triggered the Ca^2+^ transform from ER to mitochondria, resulting in mitochondrial dysfunction, and finally induced mitochondrial apoptosis. Our study shed light on a novel mechanism revealing how ER stress manipulates Ca^2+^ homeostasis to induce mitochondrial apoptosis and regulate viral proliferation.

## INTRODUCTION

Seneca Valley virus (SVV) is a non-enveloped, single-stranded, and positive-sense RNA virus. It is a unique member of the genus *Senecavirus* of the family *Picornaviridae* (1). SVV was first isolated as a cell culture contaminant in the United States in 2002 and has since been found to have oncolytic properties for the treatment of human cancers (2, 3). The first association between SVV and vesicular disease in pigs was reported in Canada in 2007 (4). Several SVV outbreaks in pigs have since been reported in many countries (5–8). Moreover, novel SVV strains with different virulence have emerged and spread throughout the Americas and Asia since 2015 (9, 10). These strains have caused severe clinical symptoms in pigs and led to the deaths of newborn piglets. This change in the pathogenicity of SVV strains has caused substantial economic losses in the trade of pigs and their products (11). Therefore, the impact of SVV on the pig industry has received increasing attention, owing to its high morbidity.

The ER, a central and multifunctional organelle, is primarily responsible for the folding, translocation, and post-translational modifications of intracellular proteins, calcium homeostasis, and redox responses in eukaryotic cells (12). However, the imbalance in endoplasmic reticulum (ER) homeostasis originates from some factors, such as viral infection and excessive protein synthesis, resulting in ER stress (13). PERK, one of the three ER transmembrane sensors, is phosphorylated to form active PERK and activate eukaryotic translation initiation factor 2 (eIF2α) to ultimately eliminate ER stress. Prolonged ER stress converts pro-survival PERK signals into pro-death signals. Additionally, ER stress can induce apoptosis through other signals, including ER-resident caspases, Bax/Bcl-2-related proteins, and ER channels (14–16). It causes the release of ER Ca^2+^, leading to Ca^2+^ influx from the cytoplasm into the mitochondria or through contact between the ER and mitochondria (17). The increase in Ca^2+^ loads in the mitochondria, exceeding a critical threshold, becomes a pro-apoptotic inducer. This changes the permeability of mitochondrial membrane potential (MMP) (18–21) and further induces mitochondrial dysfunction, which is involved in the generation of reactive oxygen species (ROS) and the synthesis of ATP (22, 23), ultimately resulting in mitochondrial apoptosis.

Owing to the continuous elevation of Ca^2+^ inside cells, viral infection-induced disturbance of Ca^2+^ homeostasis triggers apoptosis through different pathways. The porcine circovirus type 2 (PCV2) mutant, an open reading frame 3 (ORF3)-deficient strain, induces mitochondrial apoptotic responses by disrupting intracellular Ca^2+^ homeostasis and generating ROS (20). Moreover, Ca^2+^ flux and calpain-mediated activation of apoptosis-inducing factors contribute to enterovirus 71 (EV71)-induced apoptosis (24). Similarly, poliovirus infection increases cytosolic Ca^2+^ loads (25), and the transfer of the Ca^2+^ flux from the ER to the mitochondria contributes to poliovirus-mediated apoptosis (26). The activation of ER stress and the PERK pathway are involved in the pathogenicity of SVV, another member of the picornavirus family (27). Moreover, 2C or 3C proteins are responsible for SVV-mediated mitochondrial apoptosis (28). However, the relationship between ER stress and Ca^2+^ homeostasis and the effect of Ca^2+^ efflux from the ER on SVV-mediated mitochondrial apoptosis remain unknown.

This study explored the relationship between ER stress and apoptosis in SVV-infected PK-15 and BHK-21 cells. SVV-mediated ER stress and the PERK pathway led to apoptosis and the transfer of Ca^2+^ from the ER to the mitochondria. Additionally, the continuous elevation of intracellular Ca^2+^ inside cells triggered mitochondrial dysfunction, characterized by reduction of MMP and ATP, mitochondrial permeability transition pore (mPTP) opening, and ROS increase. Our results provide new insights into the mechanism of the released Ca^2+^, including ER-mediated apoptosis through crosstalk between ER stress or PERK and mitochondrial dysfunction in PK-15 and BHK-21 cells.

## RESULTS

### ER stress or PERK pathway is involved in SVV-mediated apoptosis

**Fig 1:**
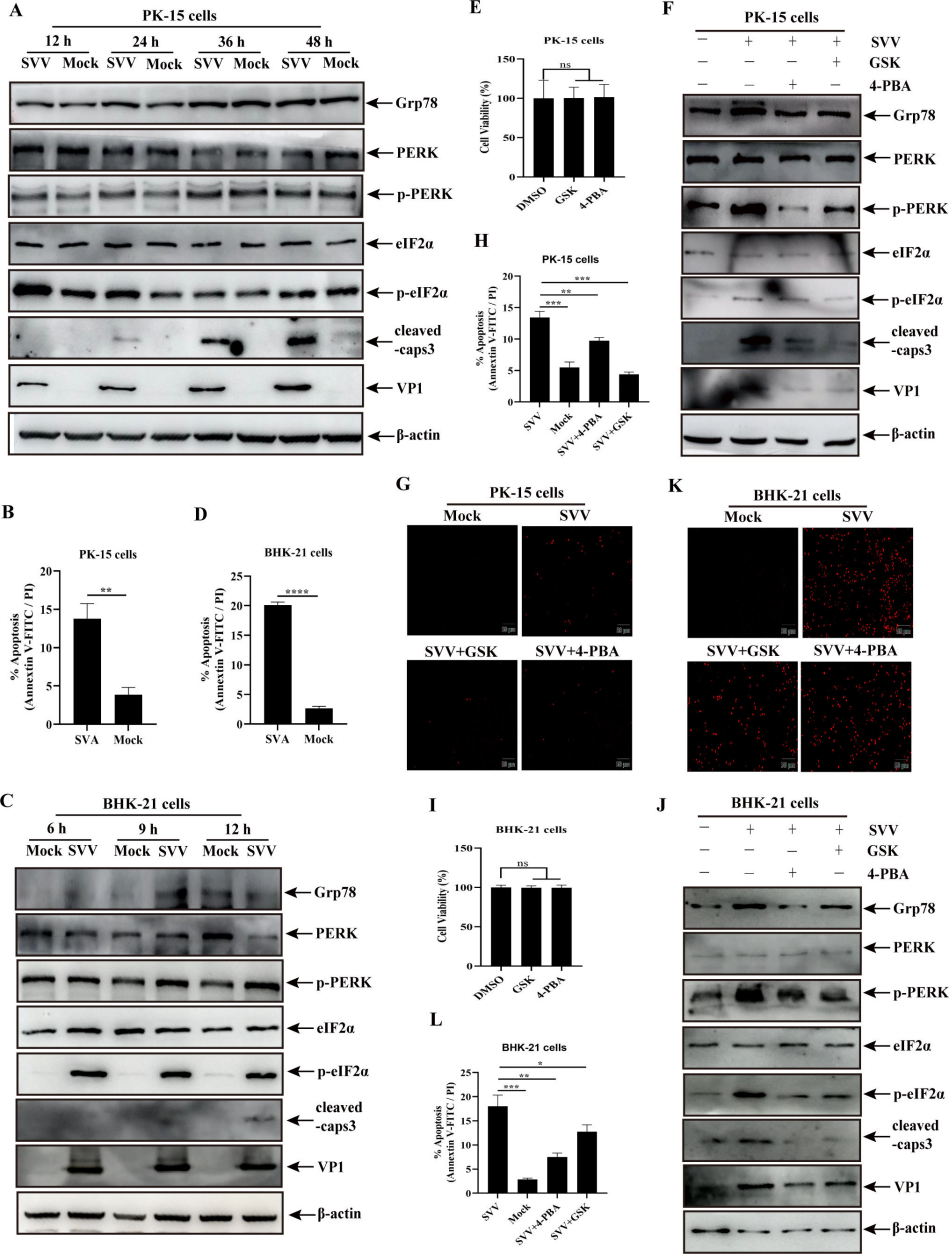
The effect of ER stress or PERK pathway on SVV-mediated apoptosis. (**A and C**) Proteins were detected by western blotting analysis with anti-Grp78, anti-PERK, anti-p-PERK, anti-eIF2α, anti-p-eIF2α, anti-cleaved-caspase3, anti-VP1, and anti-β-actin antibodies in SVV- or mock-infected PK-15 cells at 12, 24, 36, and 48 hpi (**A**) or BHK-21 cells at 6, 9, and 12 hpi (**C**). (**B and D**) Apoptosis rates (early apoptosis plus late apoptosis) in PK-15 (**B**) and BHK-21 cells (**D**) were quantified and shown in a histogram. (**E and I**) Cell viability after 4-PBA or GSK treatment in PK-15 (**E**) and BHK-21 cells (**I**) was evaluated using the CCK-8 kit. (**F and J**) PK-15 (**F**) and BHK-21 cells (**J**) were infected or uninfected with SVV in the presence or absence of 4-PBA (2 mM) or GSK (5 µM) for 24 or 9 h, followed by western blotting, as described in panel A. (**G and K**) PK-15 (**G**) or BHK-21 cells (**K**) were infected or uninfected with SVV in the presence or absence of 4-PBA or GSK and stained using a TUNEL kit, followed by detection under an immunofluorescence microscope. Red signals represent apoptotic cells. (**H and L**) Apoptosis rates in PK-15 (**H**) and BHK-21 cells (**L**) treated with 4-PBA or GSK were quantified and shown in a histogram. Data are expressed as means ± standard deviations (SDs) from three independent experiments (not significant [ns], *P* > 0.05; *P ＜ 0.05; **P ＜ 0.01; ***P ＜ 0.001; ****P ＜ 0.0001).

Our previous study showed that ER stress, PERK-eIF2α axis, and activating transcription factor 6 (ATF6) in the UPR pathway contributed to SVV replication (27). ER stress initially originates from protein aggregates-mediated endogenous imbalances in cells, especially during the viral infection process (29). In response to ER stress, UPR pathway is activated for restoring cellular protein homeostasis (30). In this study, we analyzed the expression of Grp78 (an ER stress marker) and change of the PERK-eIF2α axis in SVV-infected cells at different time points. SVV infection weakly increased Grp78 and p-PERK expression and significantly enhanced p-eIF2α expression in PK-15 cells at 12 and 24 h in PK-15 cells compared to the control groups, while no differences in the expression of the above proteins between mock- and SVV-infected cells were observed at 36 and 48 h post-infection (hpi) (Fig. 1A). Additionally, we also confirmed that SVV infection activates ER stress and the PERK pathway in BHK-21 cells as evidenced by increases in Grp78 expression at 6 and 9 h and p-PERK and p-eIF2α expression in BHK-21 cells at 9 and 12 h (Fig. 1C). These results indicated that SVV infection activated ER stress and the PERK pathway in PK-15 and in BHK-21 cells. Meanwhile, cleaved caspase-3 was initially and weakly detected at 24 and 9 hpi in PK-15 and BHK-21 cells, respectively (Fig. 1A and C), implicating a close relationship between ER stress and apoptosis in SVV-infected PK-15 and BHK-21 cells. Additionally, the expression of Bcl-2 and Bax, another two apoptosis markers, initially decreased and increased at 24 and 9 h in PK-15 and BHK-21 cells, respectively (Fig. S1A and B). Thus, these two time points (24 and 9 h) in PK-15 and BHK-21 cells were used in subsequent experiments, respectively. As shown in Fig. 1B and D and Fig. S1C and D, the number of apoptotic cells was considerably higher in SVV-infected PK-15 or BHK-21 cells than in mock-infected cells. Collectively, these results indicated that SVV induces apoptosis starting at 24 and 9 h after infection in PK-15 and BHK-21 cells, respectively.

ER stress is closely associated with apoptosis (31). 4-Phenylbutyric acid (4-PBA) and PERK activity inhibitor GSK2606414 (GSK), which are respective ER stress (20) and PERK inhibitors (20, 21), were used to analyze the effects of ER stress and the PERK pathway on apoptosis in SVV-infected cells. No significant differences were observed in cell proliferation between PK-15 and BHK-21 cells exposed to 5 µM GSK and 2 mM 4-PBA, respectively (Fig. 1E and I). PK-15 cells treated with GSK or 4-PBA were infected with SVV. As shown in Fig. 1F and Fig. S1E, GSK and 4-PBA treatment substantially reduced the activation of GRP78, p-PERK, and p-eIF2α and the expression of cleaved caspase-3 compared to the inhibitor-untreated group and attenuated SVV-mediated Bax increase and Bcl-2 decrease. The terminal deoxynucleotidyl transferase-mediated dUTP-biotin nick-end labeling (TUNEL) assay and flow cytometry showed that GSK and 4-PBA markedly reduce the number of apoptotic SVV-infected cells (Fig. 1G and H; Fig. S1G). Consistent with the PK-15 cell results, the expression of cleaved caspase-3, SVV-mediated Bax increase and Bcl-2 decrease, and the number of apoptotic cells in the BHK-21 cells were also downregulated by GSK and 4-PBA treatment (Fig. 1J through L; Fig. S1F and H). Taken together, these results indicated that ER stress and PERK pathway are involved in SVV-induced apoptosis.

### ER stress or PERK pathway is involved in SVV-mediated imbalance of Ca^2+^ homeostasis

**Fig 2:**
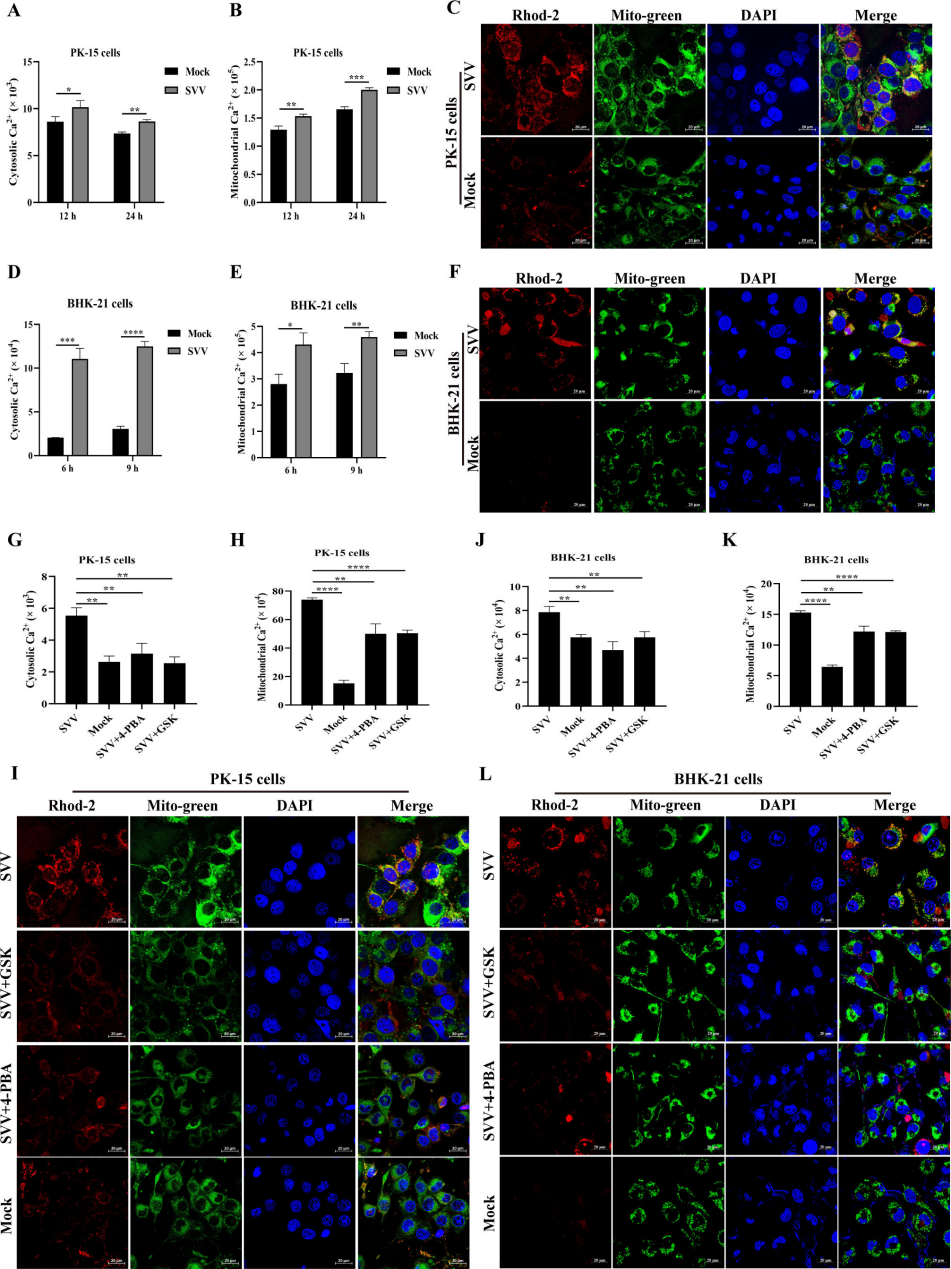
The effect of ER stress or PERK pathway on homeostasis cytoplasmic or mitochondrial Ca^2+^ in SVV-infected cells. (**A and D**) The cytoplasmic Ca^2+^ levels in PK-15 (12 and 24 hpi) (**A**) or BHK-21 cells (6 and 9 hpi) (**D**) were evaluated based on the mean values of fluo-4 fluorescence intensity and shown in a histogram. (**B and E**) The mitochondrial Ca^2+^ levels in PK-15 (12 and 24 hpi) (**B**) or BHK-21 cells (6 and 9 hpi) (**E**) were evaluated based on the mean values of rhod-2 fluorescence intensity and shown in a histogram. (**C and F**) PK-15 (**C**) or BHK-21 cells (**F**) were infected or uninfected with SVV and probed with rhod-2 (red signals), mitochondrial tracker (green signals), and DAPI (blue signals), followed by detection under an immunofluorescence microscope. (**G and J**) The levels of cytoplasmic Ca^2+^ in PK-15 (**G**) or BHK-21 cells (**H**) in the presence or absence of 4-PBA or GSK were evaluated based on the mean values of fluo-4 fluorescence intensity. (**H and K**) Mitochondrial Ca^2+^ levels in PK-15 (**H**) or BHK-21 cells (**K**) in the presence or absence of 4-PBA or GSK were evaluated based on the mean values of rhod-2 fluorescence intensity. (**I and L**) PK-15 (**I**) or BHK-21 cells (**L**) treated with 4-PBA or GSK were infected or uninfected and probed with rhod-2 (red signals), mitochondrial tracker (green signals), and DAPI (blue signals), followed by detection under an immunofluorescence microscope. Data are expressed as means ± standard deviations (SDs) from three independent experiments (*P < 0.05; **P < 0.01; ***P < 0.001; ****P , 0.0001).

The ER, a major intracellular reservoir of Ca^2+^ (32), regulates various signaling pathways such as apoptotic induction under cellular stress by transferring Ca^2+^ to the mitochondria from the ER lumen (33, 34). Thus, we investigated whether SVV infection induces ER or mitochondrial Ca^2+^ imbalance. Cells infected with SVV were incubated with fluo-4 and rhod-2 to indicate cytoplasmic and mitochondrial Ca^2+^, respectively. As shown in Fig. 2A and B, and Fig. S2A and B, Ca^2+^ levels in the cytoplasm and mitochondria were substantially increased in SVV-infected PK-15 cells compared to the mock-infected cells at 12 and 24 hpi. SVV-infected PK-15 cells were incubated with rhod-2 and MitoBright LT Green, a mitochondrial tracker, to indicate the mitochondrial Ca^2+^ content and the location of mitochondria, respectively, and then the fluorescence signals of the mitochondrial Ca^2+^ or mitochondria was observed. A significant increase was observed in mitochondria-localized Ca^2+^ in SVV-infected PK-15 cells compared to mock-infected cells at 24 h (Fig. 2C). Similarly, an increase in cytoplasmic and mitochondrial Ca^2+^ content was observed at 6 and 9 hpi (Fig. 2D and E; Fig. S2C and D), and more Ca^2+^ traced by rhod-2 entered the mitochondria at 9 hpi in the BHK-21 cells (Fig. 2F). These results indicate that SVV infection elevates cytoplasmic or mitochondrial Ca^2+^ content, disturbing Ca^2+^ homeostasis.

Disturbances in Ca^2+^ homeostasis are associated with ER stress (33). SVV infection enhanced cytoplasmic and mitochondrial Ca^2^ content in PK-15 cells compared to mock-infected cells, whereas GSK or 4-PBA treatment inhibited this increase at 24 h after SVV infection (Fig. 2G and H; Fig. S2E and F). GSK and 4-PBA treatment also inhibited SVV-mediated Ca^2+^ localization (rhod-2) in the mitochondria (MitoBright LT Green probes) of PK-15 cells at 24 hpi (Fig. 2I). Similarly, the elevation of cytoplasmic or mitochondrial Ca^2+^ concentrations mediated by SVV infection was attenuated in GSK- and 4-PBA-treated BHK-21 cells at 9 hpi (Fig. 2J through L; Fig. S2G and H). Taken together, these results indicate that ER stress plays an important role in the SVV-mediated imbalance of Ca^2+^ homeostasis.

### SVV infection induced the closed contact between ER and mitochondria

**Fig 3:**
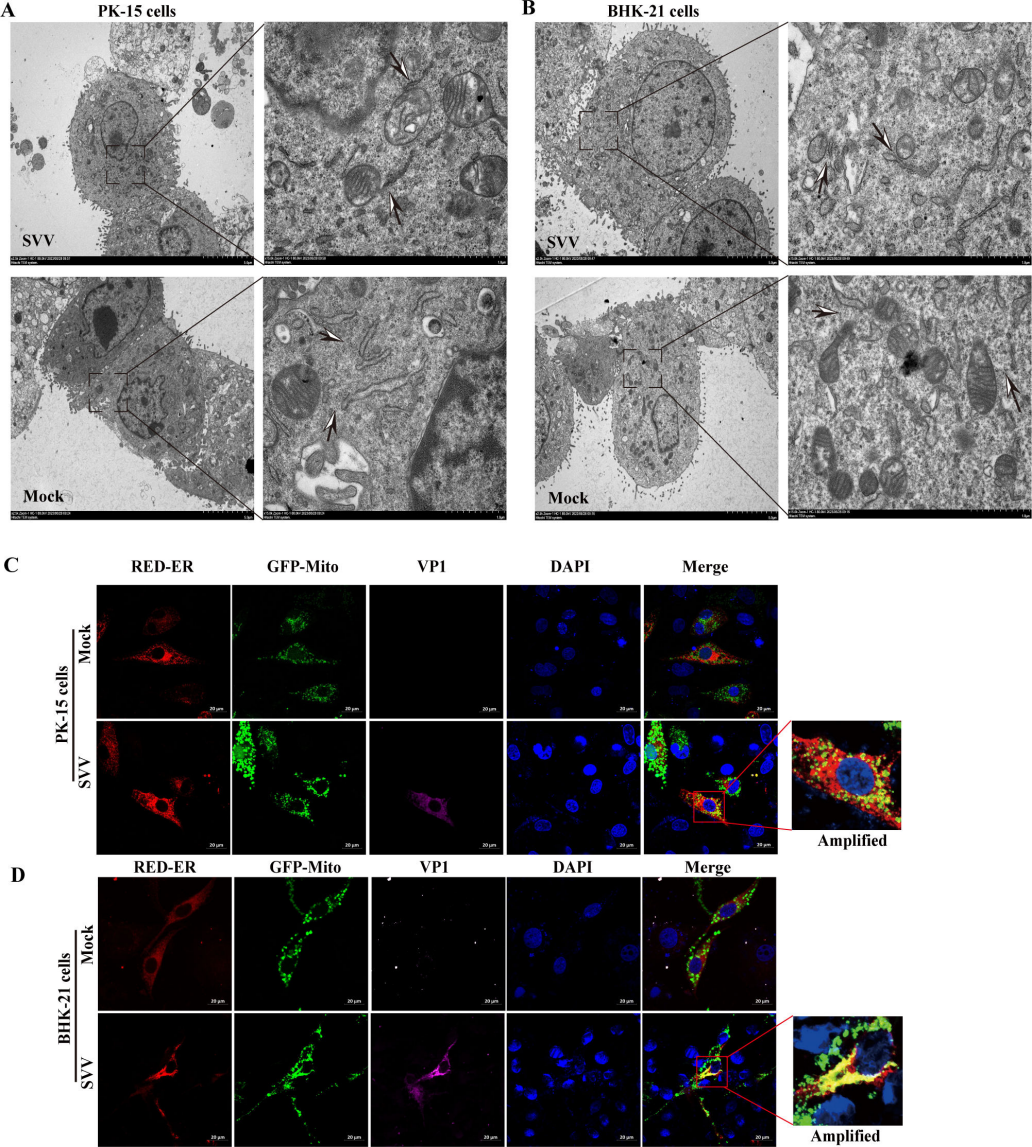
The closed contact between ER and mitochondria was detected in SVV- or mock-infected cells. (**A and B**) PK-15 (**A**) or BHK-21 cells (**B**) were infected or uninfected with SVV and electron micrographs of the connections between the ER and mitochondria were observed using transmission electron microscopy. The arrows indicate the connections between the ER and mitochondria (MAMs), whereas the black arrows indicate no connections between the ER and mitochondria. (**C and D**) PK-15 (**C**) or BHK-21 cells (**D**) co-transfected with pRED-ER (red signals) and pGFP-mitochondria (green signals) were infected or uninfected with SVV, followed by incubation with anti-VP1 antibodies (purple signals) and DAPI (blue signals). The colocalization between the ER and mitochondria was observed using a confocal immunofluorescence microscope.

The close contact between the ER and mitochondria plays an important role in the transfer of Ca^2+^ from the ER to the mitochondria (34). We continued to investigate whether the contact between the ER and mitochondria and the formation of the mitochondrion-associated ER membrane (MAM) is related to the transfer of Ca^2+^ from the ER to the mitochondria. As shown in Fig. 3A and B, SVV infection induced morphological rearrangement of the ER or mitochondria and increased the number of ER membranes near the mitochondria at 24 and 9 h PK-15 and BHK-21 cells, respectively. PK-15 and BHK-21 cells expressing RED-ER and GFP-mitochondria (GFP-mito) were then infected with SVV, respectively. The partial colocalization between red (ER) and green (mitochondria) fluorescence signals was observed in SVV-infected cells. Contrastingly, no significant colocalization was observed between the ER and mitochondria in mock-infected cells at 24 and 9 h in PK-15 and BHK-21 cells, respectively (Fig. 3C and D). These results demonstrate that the formation of MAMs and the ER-mitochondrial interface increased in SVV-infected PK-15 and BHK-21 cells, contributing to SVV-mediated Ca^2+^ transfer from the ER to the mitochondria.

### SVV-mediated Ca^2+^ increase in cytoplasm and mitochondria mainly originated from ER and promoted apoptosis

**Fig 4:**
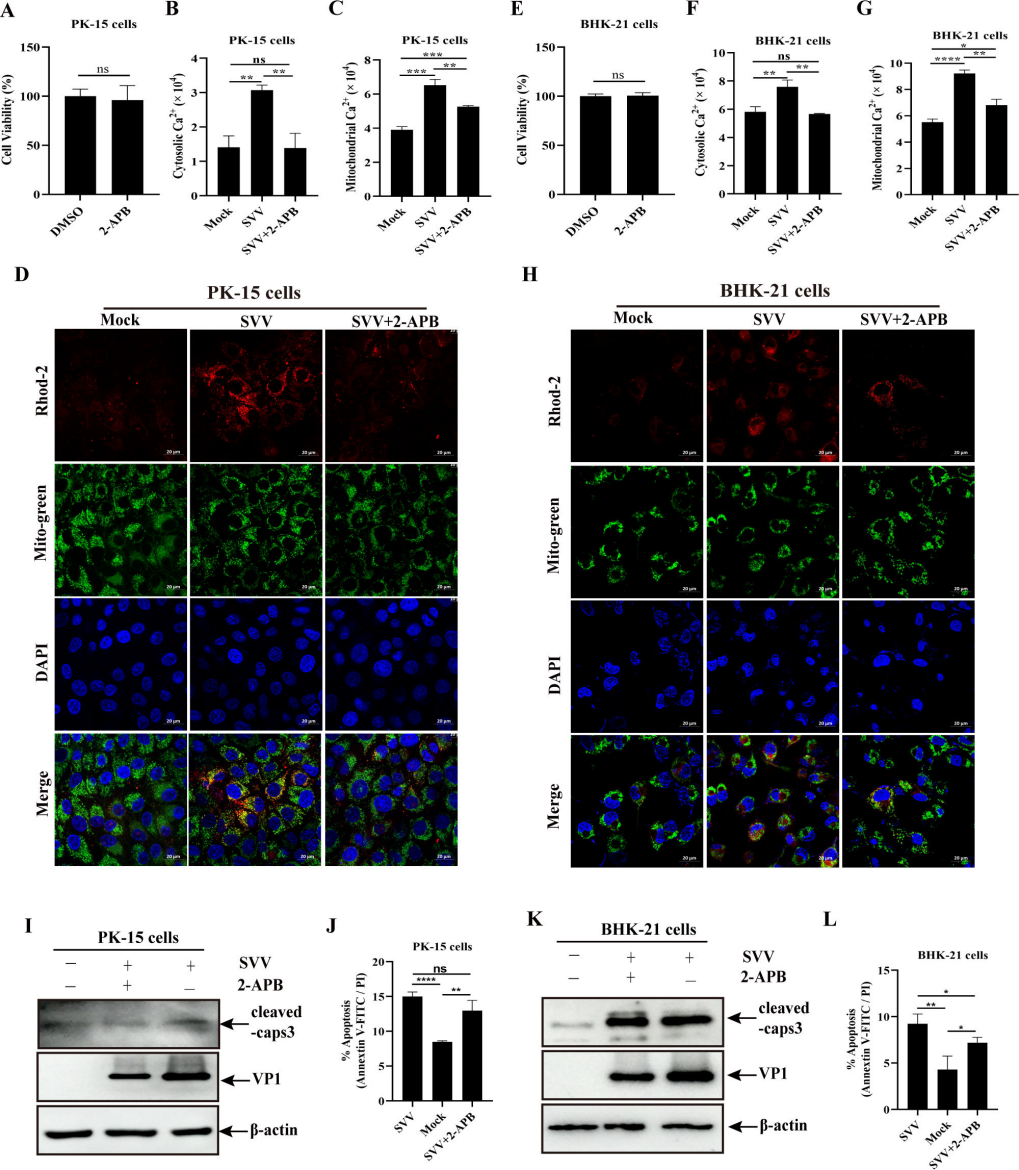
2-APB treatment attenuated increase of cytoplasmic or mitochondrial Ca^2+^and apoptosis through inhibiting ER Ca^2+^ release in SVV-infected cells. (**A and E**) Cell viability after treatment with 2-APB in PK-15 (**A**) or BHK-21 cells (**E**) was evaluated using the CCK-8 kit. (**B and F**) Cytoplasmic Ca^2+^ levels in PK-15 (**B**) or BHK-21 cells (**F**) were quantified based on the mean values of fluo-4 fluorescence intensity. (**C and G**) Mitochondrial Ca^2+^ levels in PK-15 (**C**) or BHK-21 cells (**G**) were evaluated based on the mean values of rhod-2 fluorescence intensity. (**D and H**) PK-15 (**D**) or BHK-21 cells (**H**) were probed with rhod-2 (red signals), mitochondrial tracker (green signals), and DAPI (blue signals), followed by detection under an immunofluorescence microscope. (**I and K**) Apoptosis in PK-15 (**I**) or BHK-21 cells (**K**) were analyzed by western blotting with anti-cleaved caspase-3, anti-VP1, and anti-β-actin antibodies. (**J and L**) Apoptosis rates in PK-15 (**J**) and BHK-21 cells (**L**) were quantified and shown in a histogram. Data are expressed as means ± standard deviations (SDs) from three independent experiments (not significant [ns], *P* > 0.05; *P < 0.05; **P < 0.01; ***P < 0.001; ****P < 0.0001).

The inositol 1,4,5-trisphosphate receptor (IP3R), an intracellular Ca^2+^ channel, is abundantly expressed in the ER and is found in MAMs. This receptor is activated in response to cellular stress and promotes Ca^2+^ release from the ER into the cytoplasm and mitochondria (35). Therefore, 2-aminoethoxydiphenyl borate (2-APB), an IP3R blocker of Ca^2+^ efflux from the ER, was used to identify the source of the increased Ca^2+^ in the cytoplasm and mitochondria of SVV-infected cells. Cell viability was not affected by 50 µM 2-APB (Fig. 4A and E). PK-15 cells were infected with SVV in the presence or absence of 2-APB, followed by incubation with Fluo-4 and rhod-2. As shown in Fig. 4B and C and Fig. S3A and B, 2-APB treatment reversed the SVV-mediated increase in cytoplasmic and mitochondrial Ca^2+^ compared to the 2-APB-untreated group at 24 h after SVV infection. To further confirm the effect of 2-APB on mitochondrial Ca^2+^, rhod-2 treatment was used to analyze the Ca^2+^ concentration in the mitochondria using confocal microscopy. PK-15 cells were infected with SVV in the presence or absence of 2-APB and probed with rhod-2 and Mito-Tracker Green. The tracer signals of mitochondrial Ca^2+^ were substantially localized in the mitochondria of SVV-infected PK-15 cells. However, this increase was inhibited by 2-APB treatment (Fig. 4D). Similarly, decreased cytoplasmic and mitochondrial Ca^2+^ content was observed in SVV-infected BHK-21 cells treated with 2-APB at 9 h after SVV infection (Fig. 4F and G; Fig. S3C and D). The colocalization signals between mitochondrial Ca^2+^ indicated by rhod-2 and the mitochondrial tracker were weakened by 2-APB treatment in BHK-21 cells compared to the 2-APB-untreated group (Fig. 4H). These results indicate that the ER is the main source of the increased Ca^2+^ in the cytoplasm and mitochondria of SVV-infected cells.

We explored the relationship between the Ca^2+^ released from the ER and apoptosis, as well as the expression of cleaved caspase-3 in SVV-infected cells. As shown in Fig. 4I and K, SVV infection enhanced the expression levels of cleaved caspase-3, whereas 2-APB treatment inhibited this increase and viral VP1 expression. We further analyzed the effect of 2-APB on the expression of Bcl-2 and Bax and found that 2-APB treatment attenuated SVV-mediated Bcl-2 decrease and Bax increase (Fig. S3E and F). In addition, the number of SVV-mediated apoptotic cells in the 2-APB-treated PK-15 and BHK-21 cells was lower than that in the 2-APB-untreated cells (Fig. 4J and L; Fig. S3G and H). These results indicate that the Ca^2+^ released from ER contributes to SVV-mediated apoptosis.

### The released Ca^2+^ from ER mediated by SVV infection leads to mitochondrial dysfunction and apoptosis through enhancing overload of mitochondrial Ca^2+^

**Fig 5:**
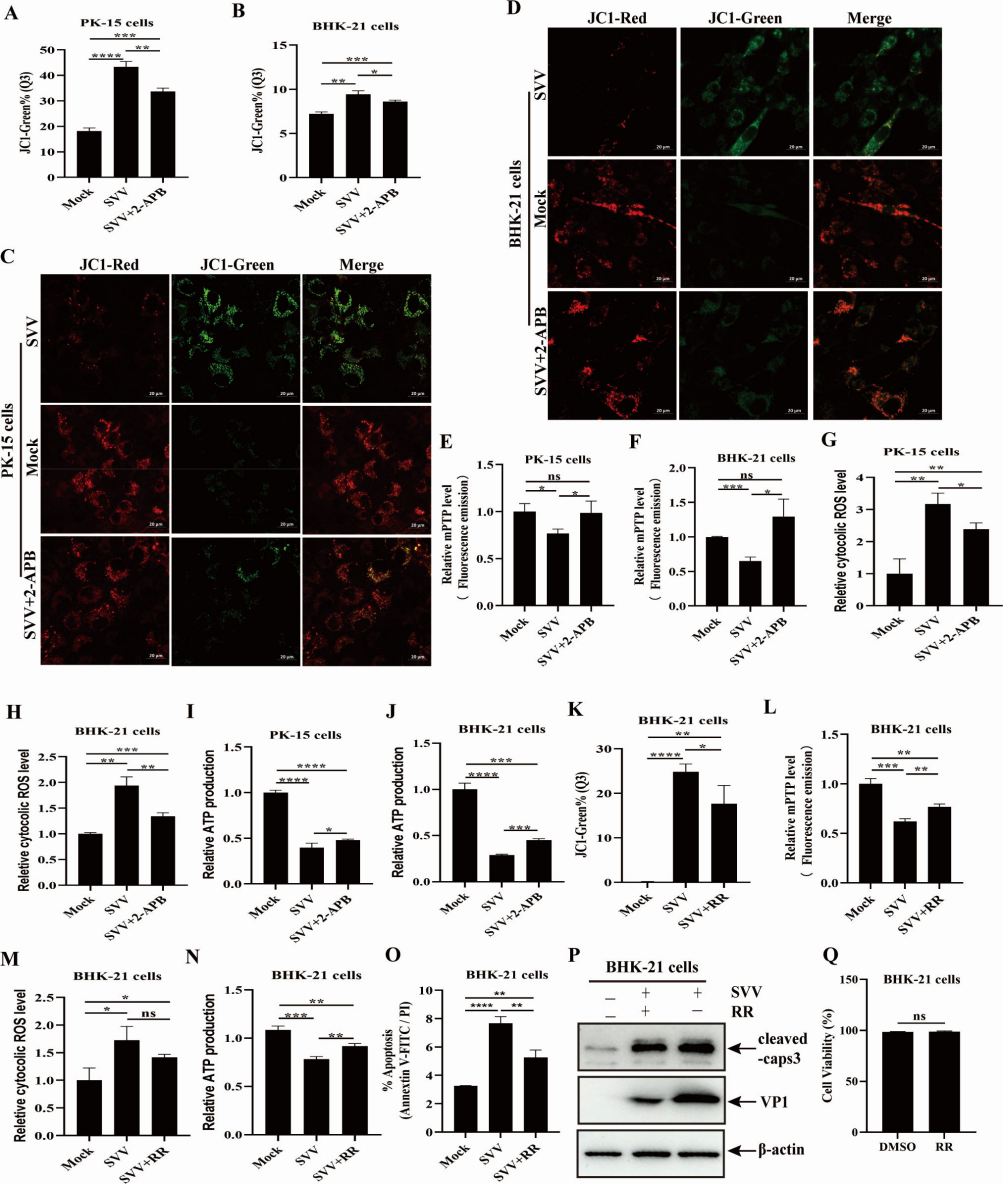
The effect of release of ER Ca^2+^ on mitochondrial dysfunction and apoptosis in SVV-infected cells. (**A and B**) JC-1 fluorescence (JC-1-green positive and JC-1-red negtive) in PK-15 (**A**) or BHK-21 cells (**B**) was evaluated based on the mean values of JC-1-green intensity. (**C and D**) PK-15 (**C**) and BHK-21 cells (**D**) were incubated with JC1, followed by observation under a confocal immunofluorescence microscope. (**E and F**) The calcein fluorescence in PK-15 (**E**) or BHK-21 cells (**F**) was evaluated based on mean values of calcein green intensity. (**G and H**) Changes in intracellular ROS levels in PK-15 (**G**) or BHK-21 cells (**H**) were quantified based on the mean values of DCFH-DA fluorescence intensity. (**I and J**) ATP production was analyzed in SVV-infected PK-15 (**I**) and BHK-21 cells (**J**) treated with 2-APB. MMP (**K**), mPTP (**L**), cytoplasmic ROS (**M**), ATP levels (**N**), and apoptotic rates (**O**) were analyzed in SVV-infected BHK-21 cells treated with RR. (**P**) Apoptosis in BHK-21 cells was analyzed by western blotting with anti-cleaved caspase-3, anti-VP1, and anti-β-actin antibodies. (**Q**) Cell viability after treatment with RR in BHK-21 cells. Data are expressed as means ± standard deviations (SDs) from three independent experiments (not significant [ns], *P* > 0.05; *P < 0.05; **P < 0.01; ***P < 0.001; ****P < 0.0001).

Mitochondrial Ca^2+^ influx, an electrogenic process, is associated with a negative MMP (34). We continued to investigate whether the mitochondrial Ca^2+^ increase affected MMP during SVV infection. PK-15 and BHK-21 cells were infected with SVV in the presence or absence of 2-APB and then probed with the fluorescent probe JC-1, the MMP was estimated using flow cytometry. As shown in Fig. 5A and B and Fig. S4A and B, SVV infection downregulated MMP in PK-15 and BHK-21 cells compared to the control groups. However, 2-APB treatment reversed the SVV-mediated MMP reduction in these cells. Furthermore, SVV-infected PK-15 and BHK-21 cells were treated with 2-APB and stained with JC-1, and changes in JC-1 fluorescence signals from red (mitochondrial location) to green (cytoplasmic location) were observed using a confocal microscope. The confocal image showed that SVV infection increased the green fluorescence signals of JC-1 and decreased the red fluorescence signals of JC-1 in PK-15 and BHK-21 cells compared to the mock-infected cells. However, 2-APB treatment attenuated this conversion of JC-1 fluorescence signals (Fig. 5C and D). These results indicate that the released ER Ca^2+^ affects MMP in SVV-infected cells.

The opening of mPTP is directly modulated by a decrease in MMP (36). To explore the effect of the Ca^2+^ release from the ER on mPTP opening, SVV-infected PK-15 and BHK-21 cells were incubated with calcein acetoxymethyl ester (Calcein AM) in the presence or absence of 2-APB, followed by flow cytometric analysis. A decrease in the fluorescence signal of calcein indicated mPTP opening. SVV infection substantially enhanced mPTP opening, characterized by the reduction of calcein fluorescence signals compared to mock-infected cells (Fig. 5E and F; Fig. S4C and D). In contrast, 2-APB treatment enhanced the calcein fluorescence signals compared to the SVV-infected cells untreated with 2-APB, which restored SVV-mediated mPTP opening. These results indicate that the SVV-mediated release of Ca^2+^ from the ER plays an important role in mPTP opening.

Oxidative stress, energy crises, and damage, as demonstrated by increased intracellular ROS and decreased ATP content, contribute to mitochondrial dysfunction (37–39). Here, we explored the effect of the SVV-mediated release of ER Ca^2+^ on intracellular ROS and ATP levels. PK-15 and BHK-21 cells infected with SVV were treated with 2-APB and probed with 2′,7′-dichlorodihydrofluorescein diacetate (DCFH-DA), an ROS indicator, followed by flow cytometric analysis. Intracellular ROS levels were markedly enhanced by SVV infection compared to the mock-infected group. Contrastingly, the SVV-mediated increase in intracellular ROS was blocked by 2-APB treatment (Fig. 5G and H; Fig. S4E and F). Reduced mitochondrial ATP is another marker of mitochondrial damage. SVV infection decreased ATP content, while this reduction was restored by 2-APB (Fig. 5I and J), suggesting that the SVV-mediated release of ER Ca^2+^ reduced ATP production. These results indicate that the SVV-mediated release of ER Ca^2+^ induced mitochondrial dysfunction, characterized by the loss of MMP and mPTP opening, increased ROS generation, and reduced ATP production.

BHK-21 cells treated with ruthenium red (RR), a blocker of mitochondrial Ca^2+^ influx, were infected with SVV and detected for MMP, mPTP opening, intracellular ROS generation, or ATP production to verify whether excessive mitochondrial Ca^2+^ plays a crucial role in SVV-mediated mitochondrial dysfunction. RR treatment attenuated SVV-mediated MMP reduction (Fig. 5K; Fig. S4G). SVV-mediated mPTP opening was inhibited in RR-treated BHK-21 cells (Fig. 5L; Fig. S4H). The regulation of intracellular ROS generation or ATP production by SVV infection was restored by RR treatment in BHK-21 cells compared to untreated cells (Fig. 5M and N; Fig. S4I). Mitochondrial Ca^2+^ overload could serve as an important initiating signal in mitochondrial apoptosis (40). As shown in Fig. 5O and P and Fig. S4J and K, SVV-induced apoptosis was inhibited by RR treatment through flow cytometry and western blotting detection. Additionally, no differences of cell viability between Mock- and RR-treated were observed (Fig. 5P). These results indicate that RR treatment suppresses SVV-induced mitochondrial dysfunction and apoptosis by blocking the influx of mitochondrial Ca^2+^.

### ER stress and the PERK pathway inhibited by chemical regents reduce mitochondrial dysfunction

**Fig 6:**
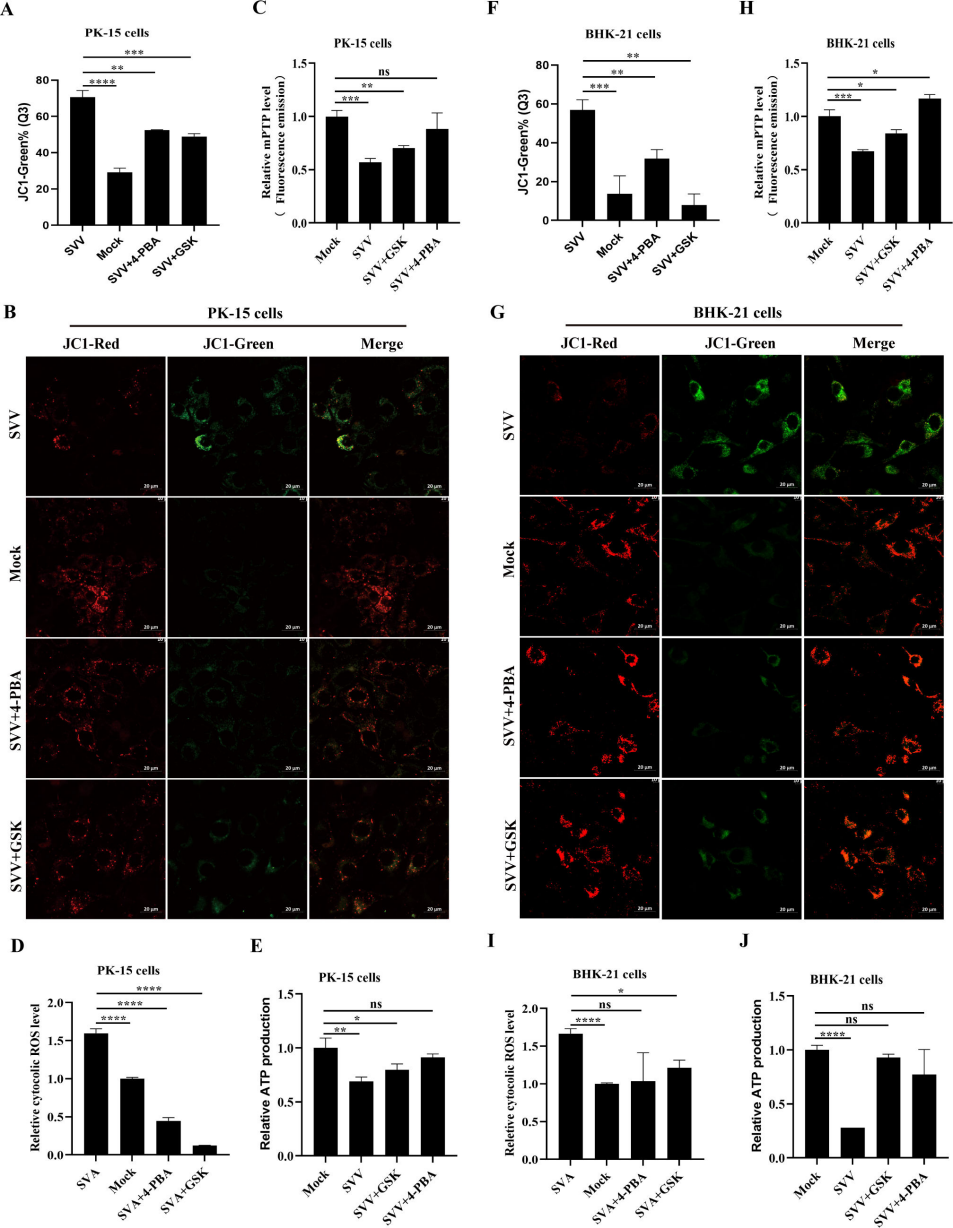
Inhibition of ER stress or the PERK pathway by 4-PBA or GSK weakened SVV-induced mitochondrial dysfunction. (**A and F**) MMP in 4-PBA- or GSK-treated PK-15 (**A**) or BHK-21 cells (**F**) was quantified based on the mean values of JC-1-green intensity. (**B and G**) PK-15 (**B**) and BHK-21 cells (**G**) were incubated with JC1, followed by observation under a confocal immunofluorescence microscope. (**C and H**) The mPTP in 4-PBA- or GSK-treated PK-15 (**C**) or BHK-21 cells (**H**) was quantified based on mean values of calcein green intensity. (**D and I**) Intracellular ROS levels in 4-PBA- or GSK-treated PK-15 (**D**) or BHK-21 cells (**I**) were quantified based on the mean values of DCFH-DA fluorescence intensity. (**E and J**) ATP production was analyzed in 4-PBA- or GSK-treated PK-15 (**E**) and BHK-21 cells (**J**). Data are expressed as means ± standard deviations (SD) from three independent experiments (not significant [ns], *P* > 0.05; *, *P* ＜ 0.05; **, *P* ＜ 0.01; ***, *P* ＜ 0.001; ****, *P* ＜ 0.0001).

The effect of ER stress and the PERK pathway on mitochondrial dysfunction in SVV-infected cells was investigated. This included MMP, mPTP, ROS, and ATP detection. PK-15 cells treated with GSK or 4-PBA were infected with SVV and probed with JC-1 for MMP detection by flow cytometry or confocal microscopy. GSK and 4-PBA treatment partially restored SVV-mediated MMP reduction (Fig. 6A; Fig. S5A) and attenuated the conversion of JC-1 fluorescence signals from red (aggregates) to green (monomers) at 24 h in SVA-infected PK-15 cells (Fig. 6B). Moreover, SVV-mediated mPTP opening was inhibited in GSK- or 4-PBA-treated PK-15 cells (Fig. 6C; Fig. S5B). The results of intracellular ROS and ATP detection were used to further evaluate mitochondrial dysfunction. As shown in Fig. 6D and E, and Fig. S5C, intracellular ROS levels and ATP content were decreased or enhanced by GSK or 4-PBA treatment in SVV-infected PK-15 cells compared to untreated cells. Similarly, the effects of GSK and 4-PBA treatment on MMP, mPTP, ROS, and ATP were evaluated in SVV-infected BHK-21 cells. Treatment with GSK or 4-PBA considerably attenuated the reduction in MMP and ATP production, mPTP opening, and the increase in intracellular ROS in SVV-infected BHK-21 cells (Fig. 6F through J; Fig. S5D through F). These results indicate that SVV-mediated activation of ER stress and the PERK pathway causes mitochondrial dysfunction by increasing ER Ca^2+^ release.

### PERK knockdown with siRNA targeting the PERK gene (siPERK) attenuates SVV-mediated apoptosis involving the disturbance of Ca^2+^ homeostasis and mitochondrial dysfunction

**Fig 7:**
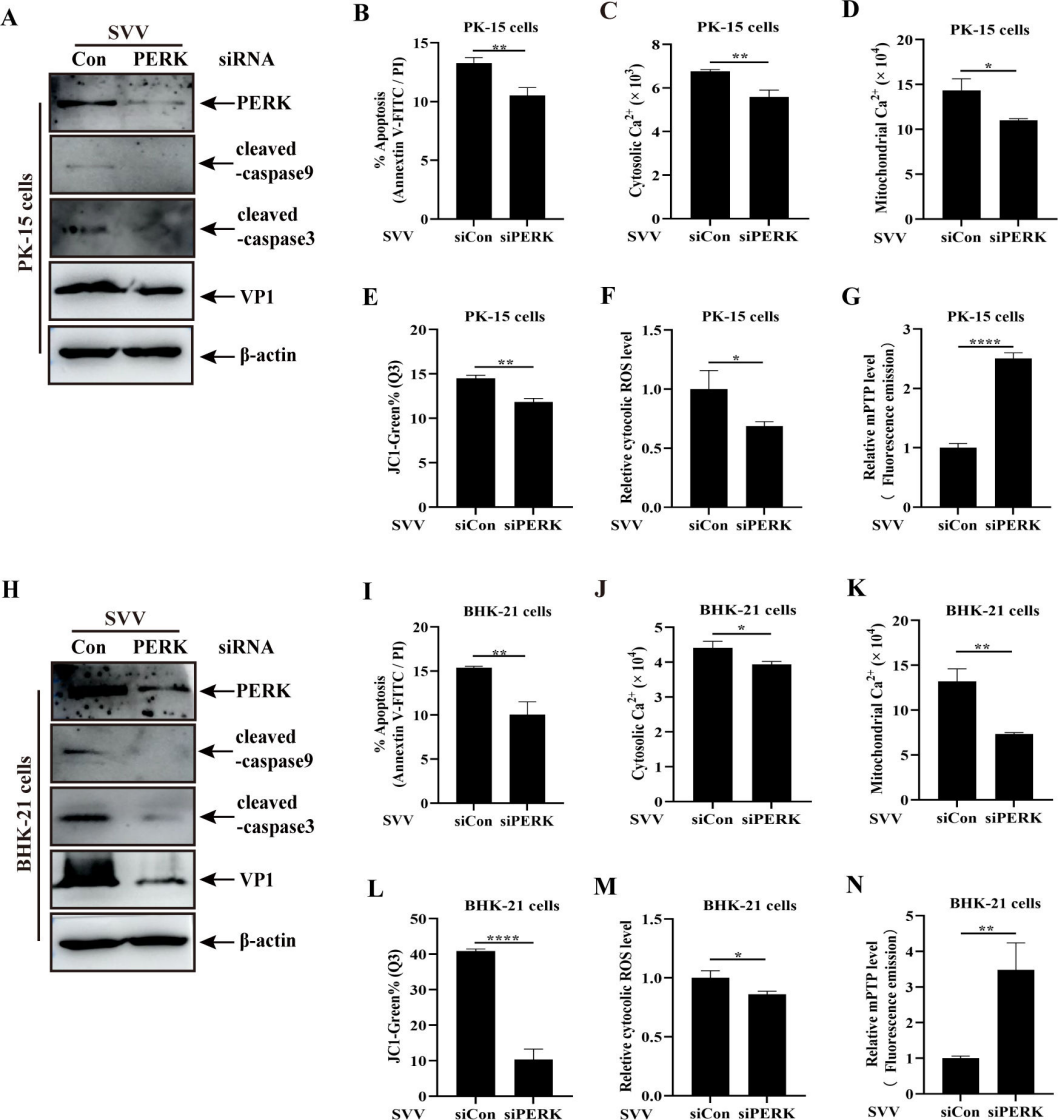
PERK knockdown reduced Ca^2+^ content in the ER and mitochondria, mitochondrial dysfunction, and apoptosis. (**A and H**) PK-15 (**A**) or BHK-21 cells (**H**) transfected with siPERK or siCon were infected with SVV, followed by western blotting with anti-PERK, anti-cleaved caspase-9, anti-cleaved caspase-3, anti-VP1, and anti-β-actin antibodies. (**B and I**) Apoptotic cell rate, (**C and J**) cytoplasmic Ca^2+^ concentration, (**D and K**) mitochondrial Ca^2+^ concentration, (**E and L**) MMP levels, (**F and M**) cytoplasmic ROS levels, and (**G and N**) mPTP were quantified in PK-15 or BHK-21 cells and shown in a histogram. Data are expressed as means ± standard deviations (SD) from three independent experiments (*, *P* ＜ 0.05; **, *P* ＜ 0.01; ****, *P* ＜ 0.0001).

The effect of PERK knockdown on SVV-induced apoptosis, Ca^2+^ imbalance, and mitochondrial dysfunction was evaluated using target-specific RNA interference to exclude the nonspecific effect of chemical reagents (GSK) on these processes. PK-15 and BHK-21 cells transfected with siPERK were infected with SVV. Western blotting demonstrated that PERK knockdown reduced the expression of cleaved caspase-9 and cleaved caspase-3 caused by SVV infection compared to the siCon group (Fig. 7A and H) and attenuated SVV-mediated Bcl-2 decrease and Bax increase (Fig. S6A and H), suggesting that the silence of PERK suppressed SVV-induced apoptosis. The inhibitory effects of siPERK on SVV-induced apoptosis were further confirmed by flow cytometry. The number of apoptotic cells induced by SVV infection was considerably reduced in siPERK-transfected PK-15 and BHK-21 cells compared to that in the siCon group (Fig. 7B and I; Fig. S6B and I). These results indicate that the PERK pathway plays an important role in SVV-induced PK-15 and BHK-21 apoptosis.

The PERK pathway contributes to Ca^2+^ homeostasis and mitochondrial dysfunction in SVV-infected cells. Thus, the effect of PERK knockdown on SVV-mediated Ca^2+^ disturbance and mitochondrial dysfunction was evaluated through siPERK. PK-15 and BHK-21 cells transfected with siPERK or siCon were infected with SVV, incubated with different probes, and analyzed for cytoplasmic or mitochondrial Ca^2+^, MMP, mPTP, and intracellular ROS using flow cytometry. PERK knockdown attenuated the increase in cytoplasmic or mitochondrial Ca^2+^ and intracellular ROS, MMP reduction, and the induction of mPTP opening (Fig. 7C through G, J through N; Fig. S6C through G, S6J through N). This would inevitably be linked to the transfer of Ca^2+^ from the ER to the mitochondria and mitochondrial Ca^2+^ overload-mediated mitochondrial dysfunction. These results confirm the involvement of the PERK pathway in the regulation of Ca^2+^ homeostasis, mitochondrial dysfunction, and apoptosis.

## DISCUSSION

The ER lumen facilitates the replication of viruses, such as the dengue and hepatitis C viruses (41, 42), and viral protein accumulation (43, 44). However, viruses or their proteins disrupt ER homeostasis through accumulating protein aggregates, induce ER stress, and initiate a UPR for alleviating this stress (29, 45), ultimately resulting in apoptosis during continuous infection. The PERK-eIF2α axis plays an important role in the activation of UPR pathway; thus, we focus on this axis on analyzing the change of UPR pathway and indirectly reflecting the activation of ER stress. This study showed that SVV infection activates ER stress and the PERK pathway. This process is characterized by a disturbance in Ca^2+^ homeostasis and the transfer of Ca^2+^ from the ER to the mitochondria, thereby triggering mitochondrial dysfunction and ultimately leading to mitochondrial apoptosis.

ER stress and apoptosis are universal cellular responses. ER stress, a result of disturbances in ER function, is common in viral infections. Our previous study confirmed that ER stress is induced during SVV infection (27). The timely response of host cells to ER stress involves the activation of UPR to reestablish equilibria (46). Cells undergo apoptosis if this reaction is not satisfied (47, 48). For example, the Japanese encephalitis virus induces apoptosis and encephalitis by activating the PERK pathway, whereas porcine circovirus type 2 induces ORF3-independent apoptosis by activating ER stress and the PERK-activating transcription factor 4 (ATF4)-CHOP axis (20, 21, 49). SVV not only has a wide range of tissue tropisms in pigs (50), but also has a broad cell tropism, such as porcine kidney epithelial (PK-15, SK6, and IBRS-2) cells, baby hamster kidney-21 (BHK-21) cells and tumor cells (11, 51). Many mechanisms of SVV infection have been elucidated on PK-15 and BHK-21 cells (27, 52, 53). Thus, PK-15 and BHK-21 cells, as two target cells for SVV infection, were selected to explore the effect of SVV infection on Ca^2+^ homeostasis, ER stress, and apoptosis. In this study, Grp78, a molecular marker of ER stress (54), was increased by SVV infection in PK-15 and BHK-21 cells compared to the control groups (Fig. 1). In the adverse effects of ER stress, the p-PERK and p-eIF2α, two crucial proteins in UPR pathway (55), were activated to minimize ER dysfunction in SVV-infected cells compared to that in Mock-infected cells, while the cleaved-caspase 3 was enhanced by the continuous infection of SVV, suggesting a possible relationship between ER stress and apoptosis in SVV-infected cells. The treatment of SVV-infected cells with an ER stress inhibitor (4-PBA) and a PERK inhibitor (GSK) confirmed this regulatory role (Fig. 1; Fig. S1). However, this study primarily investigated the precise mechanism underlying ER stress-mediated apoptosis in SVV-infected cells.

The ER is highly sensitive to viral infections (56). Moreover, Ca^2+^ homeostasis is pivotal for cell survival. Ca^2+^ is a ubiquitous intracellular signaling messenger that regulates various cellular processes, including programmed cell death (57, 58). Under cellular stress, the ER transfers Ca^2+^ to intracellular stores, particularly inside the mitochondria, where Ca^2+^ plays a crucial role in the regulation of cell death (59). In the present study, SVV infection enhanced Ca^2+^ levels in the cytoplasm and mitochondria. This is consistent with a previous report wherein PCV2-ΔORF3 infection perturbed cellular Ca^2+^ homeostasis, which was characterized by elevated cytoplasmic or mitochondrial Ca^2+^ content (20). Disruption of Ca^2+^ homeostasis in the ER is closely associated with the activation of ER stress (12). Treatment with GSK or 4-PBA inhibited the activation of the PERK pathway or ER stress (Fig. 1), further confirming the effect of these two factors on SVV-mediated disturbance of Ca^2+^ homeostasis. The SVV-mediated increase in Ca^2+^ levels in the cytoplasm and mitochondria was markedly restored in GSK- or 4-PBA-treated cells compared to the control group (Fig. 2; Fig. S2). These findings suggest that the change in cytoplasmic or mitochondrial Ca^2+^ content is related to SVV-mediated ER stress and that ER stress and the PERK pathway play an important role in cellular Ca^2+^ homeostasis.

The ER possesses the largest intracellular Ca^2+^ storage. The reduction of ER Ca^2+^ levels results in rapid Ca^2+^ accumulation in the mitochondria, which occurs through the uniporter system (60). MAMs connect the ER and the mitochondria. They consist of mitochondrial networks in the cytoplasm that dynamically interact with ER networks. These networks are involved in the transfer of Ca^2+^ from the ER to the mitochondria (61). This connection between the ER and mitochondria in cells can be observed using electron microscopy (62). Our results clarified the existence of this contact between the ER and mitochondria in the SVV-infected cells. In addition, the increased number of ER membranes near the mitochondria was further confirmed by confocal microscopy (Fig. 3). These results suggest that the formation of MAMs and the ER-mitochondrial interface caused by SVV infection may provide a basis for Ca^2+^ transfer from the ER into the mitochondria. IP3R, a ubiquitous nonselective intracellular Ca^2+^ channel, was abundantly expressed in the ER and in MAMs (63), which are responsible for the release of ER Ca^2+^ (64, 65). Treatment with 2-APB, an IP3R channel inhibitor, markedly attenuated the SVV-mediated increase in Ca^2+^ in the cytoplasm and mitochondria (Fig. 4; Fig. S3), indicating that the formation of MAMs and the ER-mitochondria interface are involved in the transfer of Ca^2+^ from the ER to the cytoplasm and mitochondria. These findings further confirmed the role of IP3R in Ca^2+^ transfer. The continuous release of ER Ca^2+^ through the IP3R channel can cause cell damage and induce apoptosis (63). For example, apoptin causes apoptosis in HepG-2 cells via Ca^2+^ imbalance and activation of the apoptotic pathway (66). In addition, ER stress and calcium flux between the ER and mitochondria are involved in virus-induced apoptosis, including poliovirus, hepatitis C virus, and PCV2 (20, 26, 54). The apoptotic level was reduced in the presence of 2-APB in the SVV-infected cells (Fig. 4; Fig. S3), suggesting that SVV induces apoptosis through the ER stress-mediated release of Ca^2+^.

Under physiological conditions, mitochondrial Ca^2+^ contents maintain stability through Ca^2+^ cycling. Stress-induced excessive Ca^2+^ concentration in the mitochondrial matrix causes mitochondrial Ca^2+^ overload and leads to mitochondrial dysfunction, including membrane de-energization, mPTP elevation, ROS production, or synthetic damage of ATP. This results in apoptosis (67, 68). The increase in ER Ca^2+^ release and elevated mitochondrial Ca^2+^ concentration in SVV-infected cells may affect normal mitochondrial function by triggering Ca^2+^ overload, as demonstrated by attenuation of SVV-mediated mitochondrial dysfunction after the inhibition of ER Ca^2+^ release by 2-APB treatment (Fig. 5; Fig. S4). Mitochondrial Ca^2+^ overload is a crucial factor in the bioenergetic crisis related to cell death and is a critical initiating signal in the intrinsic apoptotic pathways (mitochondrial apoptotic pathway) (40). This suggests that mitochondrial Ca^2+^ overload caused by ER Ca^2+^ depletion may lead to mitochondrial damage and apoptosis. Thus, RR, an inhibitor of the mitochondrial Ca^2+^ uniporter, was used to investigate whether mitochondrial Ca^2+^ overload causes mitochondrial apoptosis by preventing mitochondrial Ca^2+^ influx. RR considerably restored SVV-mediated mitochondrial dysfunction and apoptosis (Fig. 5; Fig. S4), suggesting that the SVV-induced release of Ca^2+^ from ER promotes intrinsic mitochondrial apoptosis by increasing mitochondrial Ca^2+^ influx.

The increase in cytosolic Ca^2+^ concentration and the transfer of Ca^2+^ from the ER to the mitochondria induce Ca^2+^ uptake into the mitochondria, leading to depolarization of the mitochondrial membrane and changes in membrane permeability (69). The regulatory role of ER stress in Ca^2+^ overload in the cytoplasm, mitochondria, and apoptosis prompted analysis of the relationship between ER stress and mitochondrial dysfunction. Analysis of MMP, mPTP, ROS, and ATP production demonstrated that ER stress, a positive signal of mitochondrial apoptosis, was weakened by GSK and 4-PBA and decreased mitochondrial damage (Fig. 6; Fig. S5). Exclusion of the nonspecific role of GSK in the PERK pathway using siPERK further confirmed that ER stress and the PERK pathway are involved in the regulation of Ca^2+^ homeostasis and mitochondrial apoptosis in SVV-infected cells (Fig. 7; Fig. S6).

**Fig 8:**
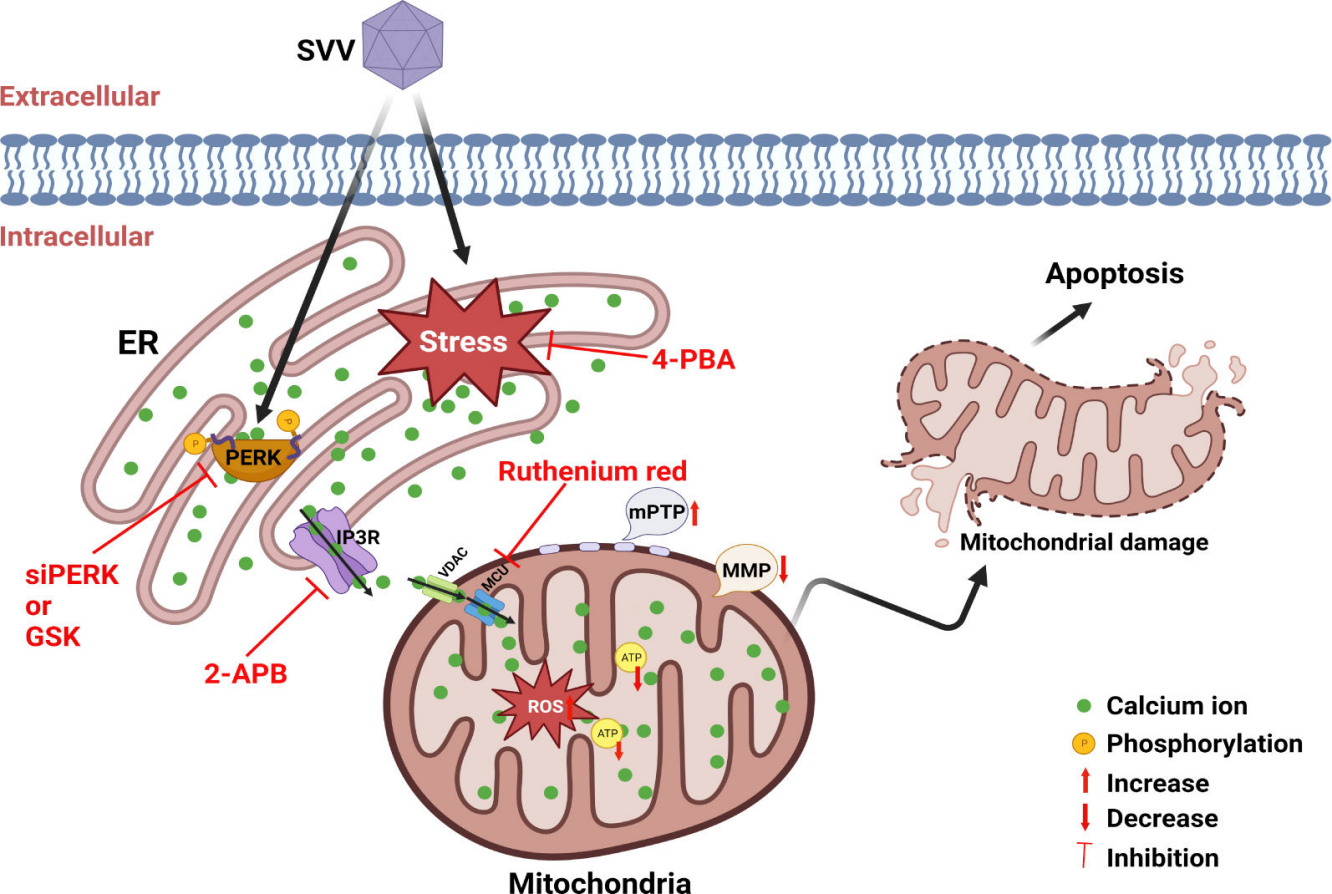
The proposed model for ER stress- or PERK pathway-mediated mitochondrial apoptosis via the transfer of Ca^2+^ from the ER to the mitochondria in SVV-infected cells. SVV infection induces ER stress and activates the PERK pathway, triggering the transfer of Ca^2+^ from the ER to the cytoplasm and mitochondria. Overload of mitochondrial Ca^2+^ leads to mitochondrial dysfunction, characterized by the loss of MMP, mPTP opening, increased ROS generation, and ATP reduction, which results in mitochondrial apoptosis. Treatment of SVV-infected cells with 2-APB (an IP3R blocker of Ca^2+^ efflux from the ER), 4-PBA (an ER stress inhibitor), GSK (a PERK pathway inhibitor), and RR (a blocker of Ca^2+^ influx into the mitochondria) suppressed SVV-induced apoptosis by inhibiting the transmission of Ca^2+^ and attenuating mitochondrial dysfunction and apoptosis.

To our knowledge, this study is the first to demonstrate the involvement of the ER stress-mediated imbalance of Ca^2+^ homeostasis in SVV-induced apoptosis. This study further confirmed that the Ca^2+^ released from the ER is transported into the mitochondria to trigger mitochondrial dysfunction, as evidenced by reducing of MMP and ATP production and mPTP and ROS, and finally result in mitochondrial apoptosis and facilitate SVV replication (Fig. 8). This comprehensive understanding of the critical roles of Ca^2+^ and its transfer pathway in the ER stress-mediated apoptosis of SVV-infected cells, and the underlying mechanisms and processes, will provide new approaches for preventing and controlling various diseases based on ER stress.

## MATERIALS AND METHODS

### Cells, viruses, antibodies, and chemical reagents

PK-15 or BHK-21 cells were cultured in high glucose Dulbecco’s modified Eagle’s medium (DMEM, Gibco; 41965039) with penicillin, streptomycin, and 5% or 10% fetal bovine serum (FBS, Sigma; F9665) inside a 5% CO_2_ incubator at 37°C. SVV CHhb17 strain preserved in our laboratory was used in this study. Mouse monoclonal anti-SVV VP1 antibody was produced in our laboratory and other antibodies were purchased commercially and included: rabbit polyclonal anti-PERK antibody (Abcam; ab79483), mouse monoclonal anti-β-actin antibody (Sigma; A5441), rabbit polyclonal anti-p-PERK antibody (Abcam; ab156919), mouse monoclonal anti-eIF2α antibody (Abcam; ab5369), rabbit monoclonal anti-p-eIF2α antibody (Abcam; ab32157), rabbit monoclonal anti-cleaved caspase3 antibody (Beyotime; AC033), rabbit anti-cleaved caspase9 antibody (ABclonal; A22672), rabbit anti-Bax antibody (ABclonal; A15646), rabbit anti-Bcl-2 antibody (ABclonal; A0208), horseradish peroxidase (HRP)-conjugated anti-rabbit or -mouse secondary antibodies (Sigma; A0545 or A9044), and Alexa Fluor 647-labeled goat anti-mouse IgG (A21236). The chemical reagents were purchased commercially and included: GSK (Santa Cruz; 1337531-89-1), 4-PBA (Selleck; S3592), 2-APB (Selleck; S6657), and RR (MCE; HY-103311).

### Virus infection

PK-15 or BHK-21 cells with a confluence of approximately 90% were pretreated with various concentrations of chemical reagents (5 µM GSK, 2 mM 4-PBA, 50 µM 2-APB, and 20 µM RR) for 1 h or transfected with siPERK or siCon for 24 h at 37°C and then infected with SVV at a multiplicity of infection (MOI) of 1 (PK-15 cells) and 5 (BHK-21 cells) at 37°C, respectively. The cells were incubated for the indicated times and further analyzed according to the different experimental requirements.

### Cell viability assay

The effects of various chemical reagents on cell viability were evaluated using a Cell Counting Kit-8 (CCK-8) (Abbkine; BMU106-CN). PK-15 or BHK-21 cells treated with various concentrations of chemical reagents were processed with the corresponding reagents of the CCK-8 kit, and cell viability was measured at specific time points according to the manufacturer’s protocols.

### Western blotting

The cells were processed with radioimmunoprecipitation assay lysis buffer (RIPA) (Beyotime; P0013B) for the whole-cell lysates, and the supernatant proteins in lysates were quantified using a bicinchoninic acid protein assay kit (Beyotime; P0011). About 20 µg of extracted proteins was analyzed by sodium dodecyl sulfate-polyacrylamide gel electrophoresis and transferred to nitrocellulose membranes (Pall; 66458), followed by blocking with 5% skim milk at room temperature for 2 h. The membranes were incubated with various primary antibodies and corresponding HRP-conjugated secondary antibodies for 2 h, and subsequently observed using a Super Signal West Pico PLUS Chemiluminescent Substrate Kit (Thermo; 34580) in a chemiluminescence apparatus (Amersham, USA).

### Measurement of apoptosis

For the annexin-V-FITC and PI assays using flow cytometry, PK-15 or BHK-21 cells infected with SVV in the presence or absence of GSK, 4-PBA, siPERK, or siCon were processed for the indicated time points and then stained with annexin-V-FITC and PI (Beyotime; C1062L) for 20 min at room temperature under the dark. More than 2 × 10^4^ cells were evaluated using a CytoFLEX flow cytometer (Beckmann, Germany) to identify the different cell populations.

For the TUNEL assay using an immunofluorescence, PK-15 or BHK-21 cells infected with SVV in the presence or absence of GSK or 4-PBA were fixed with the precooled 4% paraformaldehyde solution for 30 min at the room temperature, followed by permeabilizing with 0.2% Triton X-100 for 15 min on ice. After washing, the cells were incubated with BrightRed TdT labeling Mix (Vazyme; A113-01) according to the manufacturer’s protocol. Fluorescent images were obtained using an immunofluorescence microscope (Olympus IX73, Florida, USA).

### Measurement of cytosolic or mitochondrial Ca^2+^ levels

For the cytosolic Ca^2+^ or mitochondrial Ca^2+^ assay using flow cytometry, PK-15 or BHK-21 cells grown to approximately 80–90% confluence were infected with SVV in the absence or presence of various chemical reagents, siPERK, or siCon and then trypsinized, washed, and resuspended in Hanks’ balanced salt solution (HBSS) (Beyotime; C0218). The resuspended cells were probed with 5 µM fluo-4/acetoxy methyl (AM) (Beyotime; S1060) or 5 µM Rhod-2/AM (MedChemExpress; HY-D0989) for 30 min at 37°C under the dark, followed by measuring using a CytoFLEX flow cytometer.

For the mitochondrial Ca^2+^ assay using a confocal immunofluorescence, PK-15 or BHK-21 cells grown to approximately 80–90% confluence were infected with SVV in the absence or presence of various chemical reagents and then probed with 0.1 µM MitoBright LT Green (Dojindo; MT10) and 5 µM Rhod-2/AM (MedChemExpress; HY-D0989) for 30 min at 37°C under the dark. After washing, the cells were incubated with 4′,6-diamidino-2-phenylindole (DAPI) (Sigma; D9542), followed by observing using a confocal immunofluorescence microscope (Leica TCS SP8 STED, Weztlar, Germany).

### Measurement of cytosolic ROS, MMP, and mPTP levels

For cytosolic ROS, MMP, and mPTP assay using flow cytometry, PK-15 or BHK-21 cells infected with SVV in the absence or presence of various chemical reagents, siPERK or siCon were trypsinized, washed, and resuspended in specific buffer. The resuspended cells were stained with DCFH-DA (Beyotime; S0033S), JC-1 (Beyotime; C2006), or Calcein AM (Beyotime; C2009) for 20–30 min at 37°C under the dark, respectively. After washing, the cells labeled with different probes were measured using a CytoFLEX flow cytometer.

For MMP assay using flow cytometry or a confocal immunofluorescence, PK-15 or BHK-21 cells infected with SVV in the absence or presence of various chemical reagents were washed and incubated with JC-1 for 20 min at 37°C under the dark. After washing, the cells were detected using flow cytometry or incubated with DAPI and then observed in a confocal immunofluorescence microscope. When the MMP is high, JC-1 presents polymer form (red fluorescence) in the matrix of mitochondrial under mitochondrial potential. When the cells are subjected to the stress factors, the JC-1 is transformed into monomer form (green fluorescence) and then exists in the cytoplasm under the low MMP (70). Therefore, the reduction of MMP can be determined according to the increase of green fluorescence, which is an important sign of apoptosis.

### Measurement of ATP production

For ATP assay using a luminometer, PK-15 or BHK-21 cells infected with SVV in the absence or presence of various chemical reagents or siPERK were lysed and centrifuged, and then the ATP levels in supernatants were measured using the enhanced ATP assay kit (Beyotime; S0027) according to the manufacturer’s protocol.

### The detection of contact between ER and mitochondria

PK-15 or BHK-21 cells transfected with pRed-ER or GFP-mito plasmids were infected with SVV. After washing, the cells were incubated with DAPI and then observed using a confocal immunofluorescence microscope.

### Transmission electron microscopy

PK-15 or BHK-21 cells incubated with SVV were collected, fixed, and processed. Ultrathin sections of cells were examined using a Hitachi H-7500 transmission electron microscope (Hitachi Ltd., Tokyo, Japan). The localization of ER and mitochondria was observed using immunoelectron microscopy.

### siRNA transfection

siRNAs (sense, 5′-GUAGCUGGAACGACAUUAATT-3′; antisense, 5′-UUAAUGUCGUUCCAGCUACTT-3′) targeting the PERK gene were designed and transfected into PK-15 or BHK-21 cells using Lipofectamine RNAiMAX (Invitrogen; 13778-150) according to the manufacturer’s protocol. The cells were infected with SVV in the absence or presence of various probes, followed by subjecting to western blotting or labeling for flow cytometric analysis as described above.

### Statistical analysis

Statistical differences for all data were determined by one-way analysis of variance or Student’s *t* test using GraphPad Prism 9.0 software (GraphPad Software, CA, USA). Statistical significance was set at *P*＜0.05.

## Data Availability

All data sets generated for this study are within the paper.

## SUPPLEMENTAL MATERIAL

Supplemental figures jvi.02177-24-s0001.pdf Figures S1 to S6.

ASM does not own the copyrights to Supplemental Material that may be linked to, or accessed through, an article. The authors have granted ASM a non-exclusive, world-wide license to publish the Supplemental Material files. Please contact the corresponding author directly for reuse.
