# Supplementary material for: Seneca Valley virus induces mitochondrial apoptosis by activating ER stress or the PERK pathway based on Ca2+ transfer from ER to mitochondria
Source: J Virol. 2025 Feb 6;99(3):e02177-24. doi: 10.1128/jvi.02177-24 (PMC11915807; doi:10.1128/jvi.02177-24)
Supplement: Supplemental figures — Figures S1 to S6. [file jvi.02177-24-s0001.pdf]

SUPPLEMENTARY FIGURES and FIGURE LEGENDS

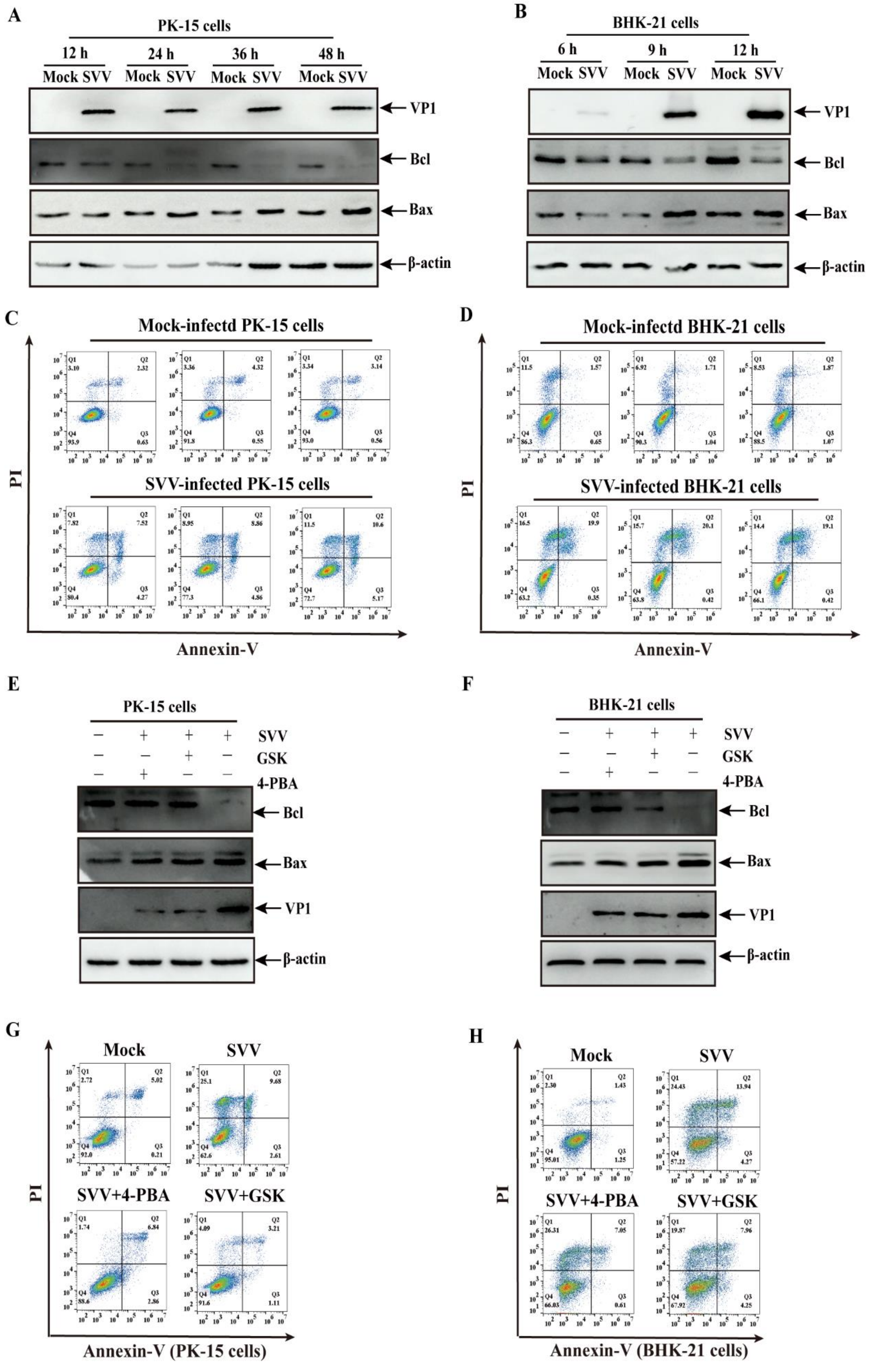

Figure S1. Flow cytometric analysis for apoptosis in SVV-infected cells treated with 4-PBA or GSK. (A and B) Proteins were detected by western blotting analysis with anti-Bax, anti-Bcl-2, anti-VP1, and anti-β-actin antibodies in SVA- or mock-infected PK-15 cells at 12, 24, 36, and 48 hpi (A) or BHK-21 cells at 6, 9, and 12 hpi (B). (C and D) PK-15 (C) and BHK-21 cells (D) were infected or uninfected with SVV and stained with annexin V-FITC and PI for flow cytometric analysis. (E and F) PK-15 (E) and BHK-21 cells (F) were infected or uninfected with SVA in the presence or absence of 4-PBA (2 mM) or GSK (5 μM) for 24 or 9 h, followed by western blotting, as described in Fig. S1A. (G and H) PK-15 (G) and BHK-21 cells (H) were infected or uninfected with SVV in the presence or absence of 4-PBA or GSK, followed by flow cytometric analysis. Apoptotic cells come from two parts of early apoptosis (annexin V positive and PI negative) and late apoptosis (annexin V positive and PI positive).

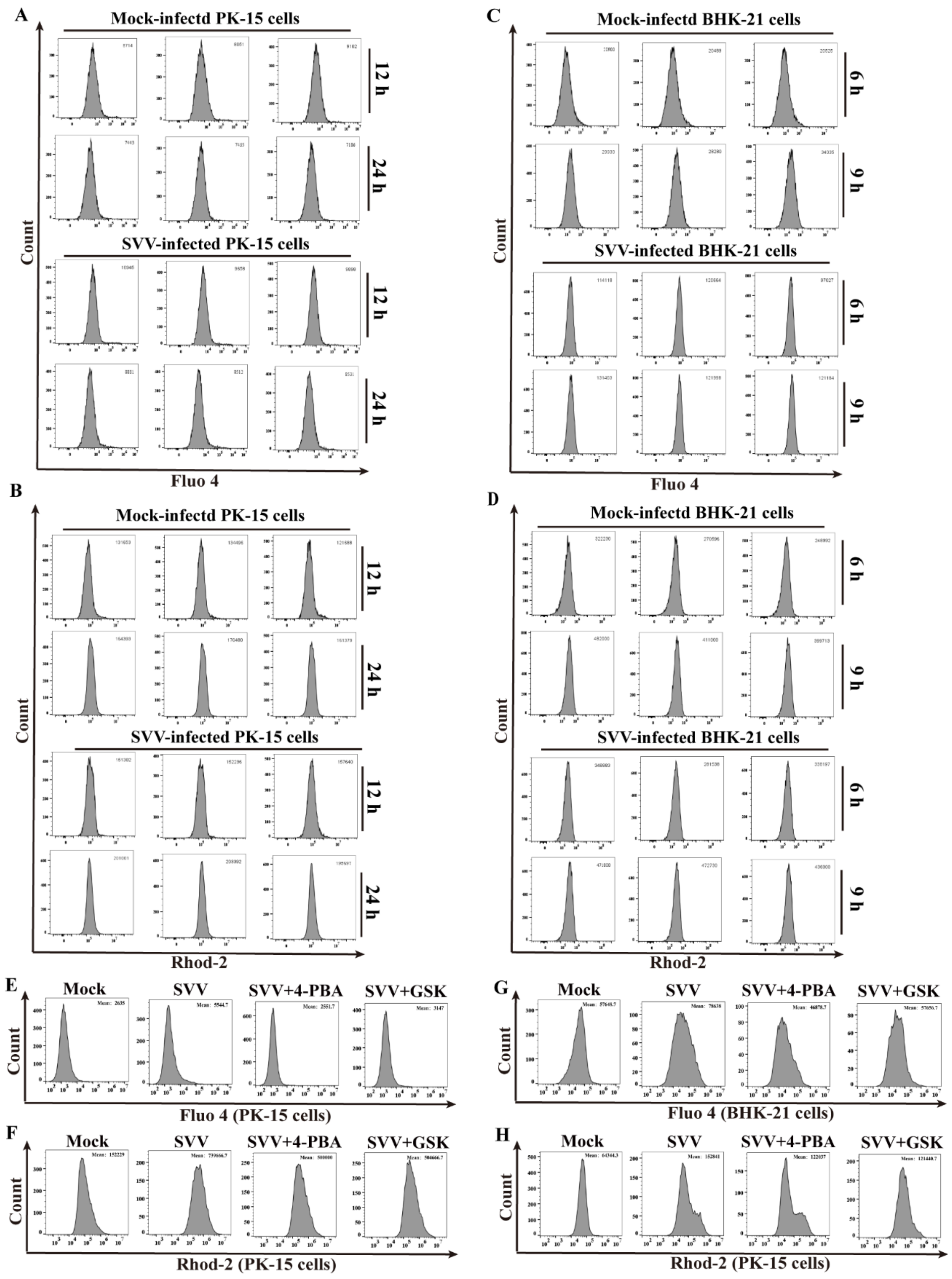

Figure S2. Flow cytometric analysis for cytoplasmic or mitochondrial Ca^{2+} in SVV-infected cells treated with 4-PBA or GSK. (A and C) Flow cytometric analysis for cytoplasmic $Ca^{2+}$with 5 μM fluo-4 in the PK-15 cells (12 and 24 hpi)- (A) or BHK-21 cells (6 and 9 hpi)- (C) infected or uninfected with SVV. (B and D) Flow cytometric analysis for mitochondrial Ca^{2+} with 5 μM rhod-

2 in the PK-15 cells (12 and 24 hpi)- (B) or BHK-21 cells (6 and 9 hpi)- (D) infected or uninfected with SVV. (E and G) PK-15 (E) or BHK-21 cells (G) were infected or uninfected with SVV in the presence or absence of 4-PBA or GSK and probed with 5 μM fluo-4, followed by flow cytometric analysis. (F and H) PK-15 (F) or BHK-21 cells (H) were infected or uninfected with SVV in the presence or absence of 4-PBA or GSK and probed with 5 μM rhod-2, followed by flow cytometric analysis.

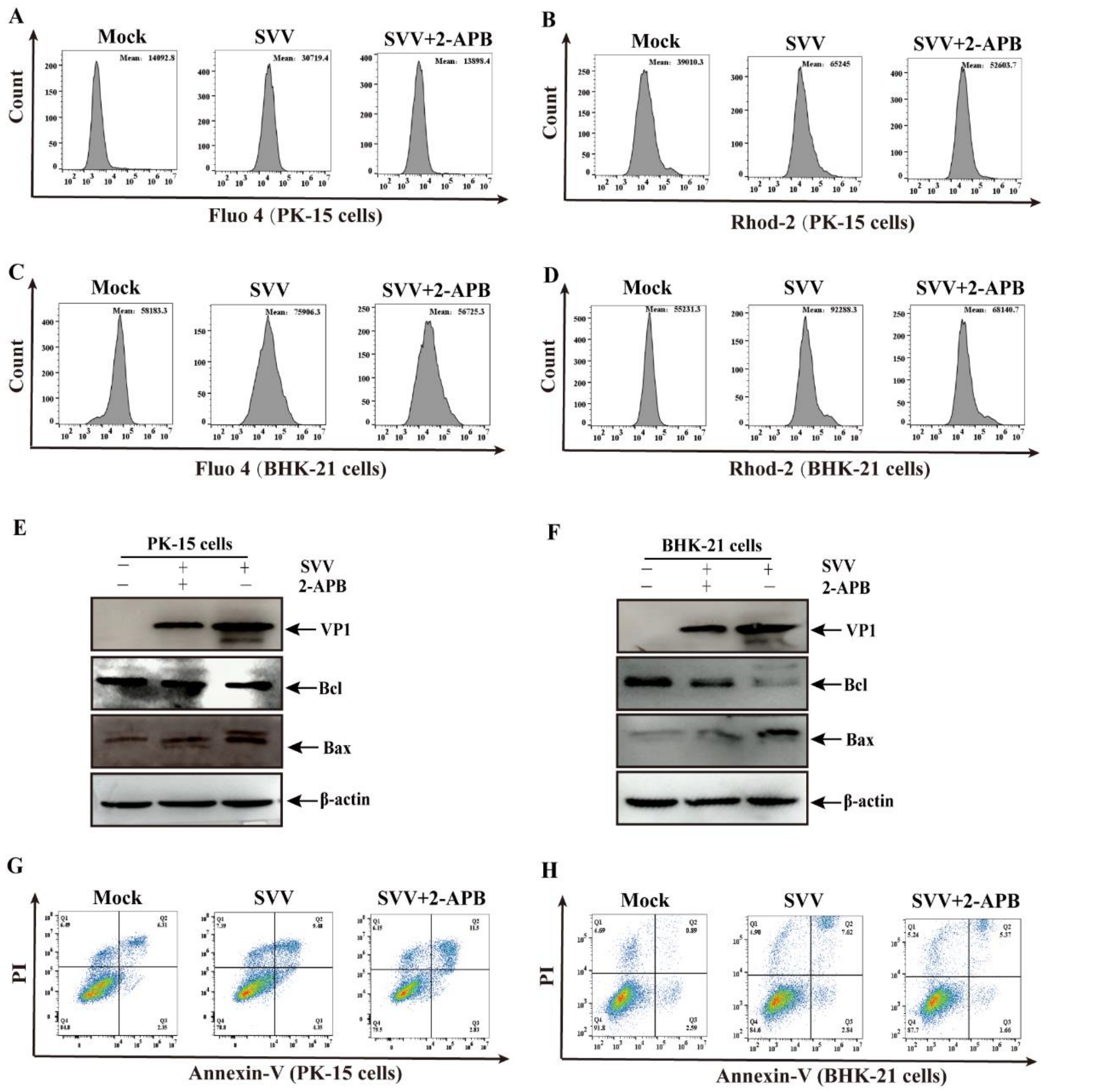

Figure S3. Flow cytometric analysis for cytoplasmic or mitochondrial Ca^{2+} and apoptosis in SVV-infected cells treated with 2-APB. (A and C) PK-15 cells- (A) or BHK-21 cells -(C) infected with SVV were treated with 2-APB and probed with fluo-4, followed by flow cytometric analysis. (B and D) PK-15 cells- (B) or BHK-21 cells -(D) infected with SVV were treated with 2-APB and probed with rhod-2, followed by flow cytometric analysis. (E and F) PK-15 (E) and BHK-21 cells (F) were infected or uninfected with SVA in the presence or absence of 2-APB (50 µM) for 24 or 9 h, followed by western blotting, as described in Fig. S1A. (G and H) PK-15 cells- (G) and BHK-21 cells- (H) infected with SVV were treated with 2-APB and stained with annexin V-FITC and PI for flow cytometric analysis.

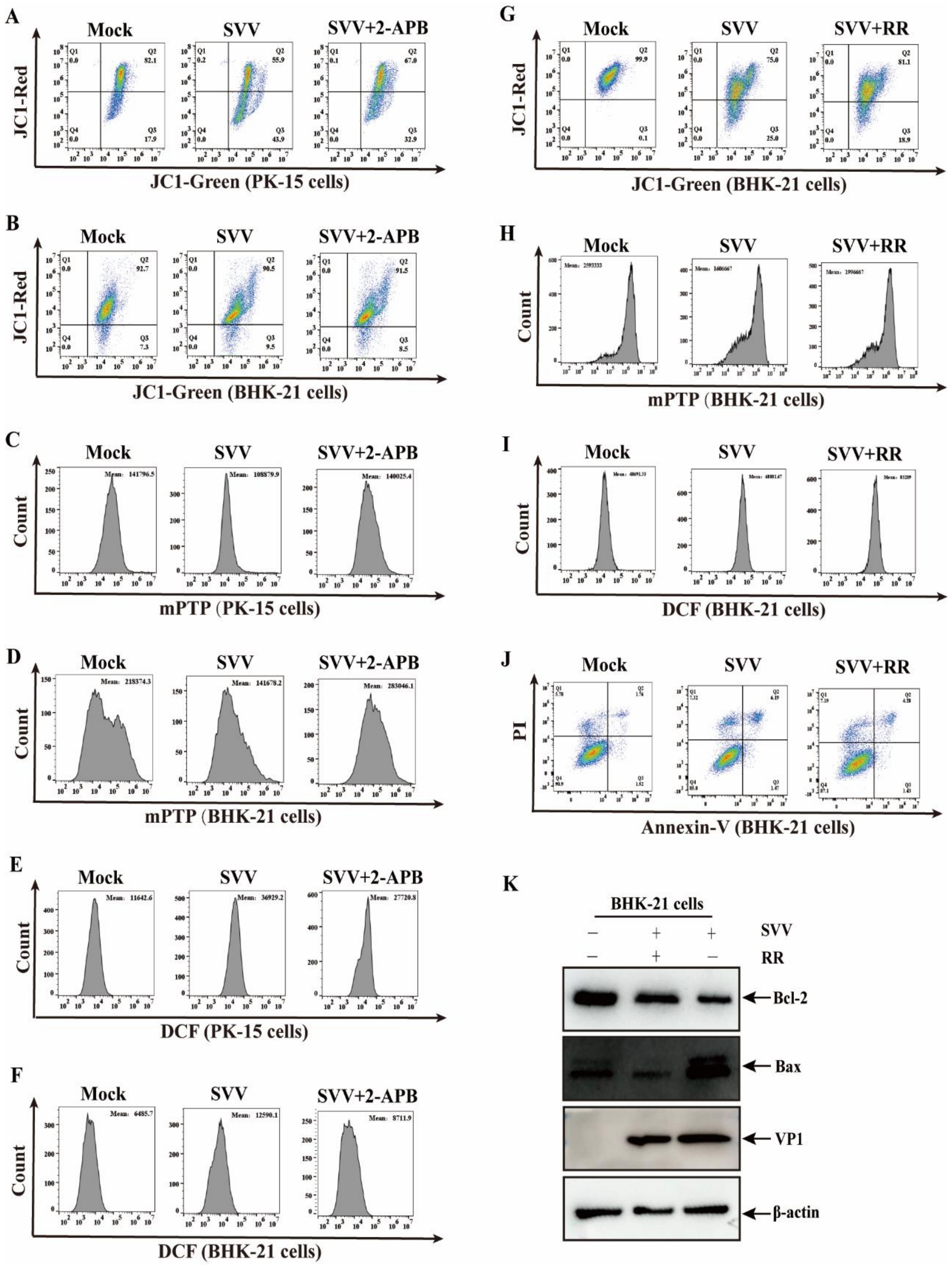

Figure S4. Flow cytometric analysis for mitochondrial dysfunction in SVV-infected cells treated with 2-APB or RR. (A and B) PK-15 cells- (A) or BHK-21 cells- (B) treated with 2-APB were infected SVV in and probed with JC-1, followed by flow cytometric analysis. (C and D) PK-

15 cells- (C) or BHK-21 cells- (D) treated with 2-APB were infected SVV in and probed with with calcein, followed by flow cytometric analysis. (E and F) PK-15 cells- (E) or BHK-21 cells- (F) treated with 2-APB were infected SVV in and probed with with DCFH-DA, followed by flow cytometric analysis. Flow cytometric analysis for MMP (G), mPTP (H), ROS (I), and apoptosis (J) in SVV-infected BHK-21 cells treated with RR (20 μM). (K) BHK-21 cells were infected or uninfected with SVA in the presence or absence of RR (20 μM) for 9 h, followed by western blotting, as described in Fig. S1A.

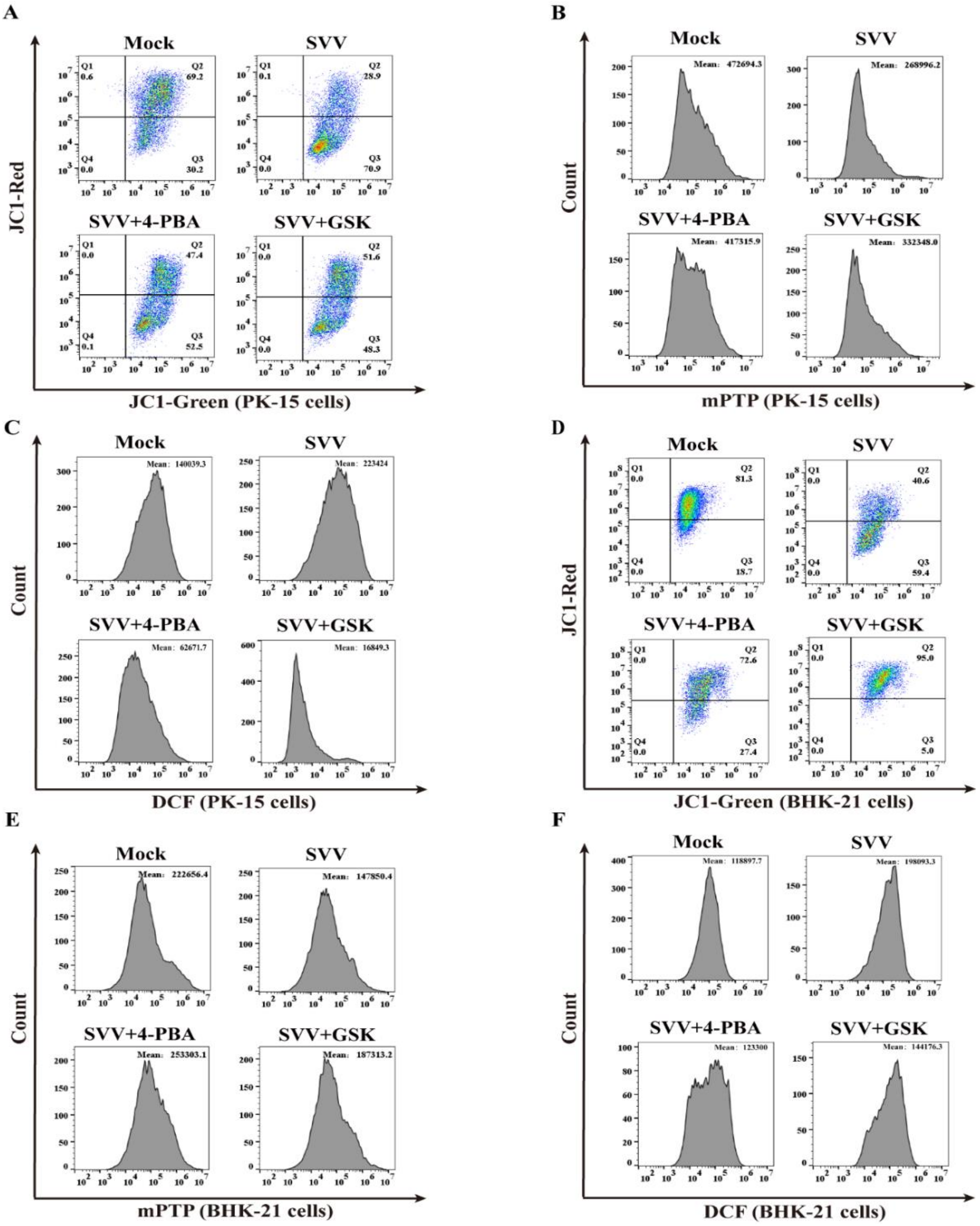

Figure S5. Flow cytometric analysis for mitochondrial dysfunction in SVV-infected cells treated with 4-PBA or GSK. (A and D) PK-15 cells- (A) or BHK-21 cells- (D) treated with 4-PBA or GSK were infected SVV in and probed with JC-1, followed by flow cytometric analysis for MMP. (B and E) PK-15 cells- (B) or BHK-21 cells- (E) treated with 4-PBA or GSK were infected SVV in and probed with with calcein, followed by flow cytometric analysis for mPTP. (C and F) PK-15 cells- (C) or BHK-21 cells- (F) treated with 4-PBA or GSK were infected SVV in and probed with with DCFH-DA, followed by flow cytometric analysis for ROS.

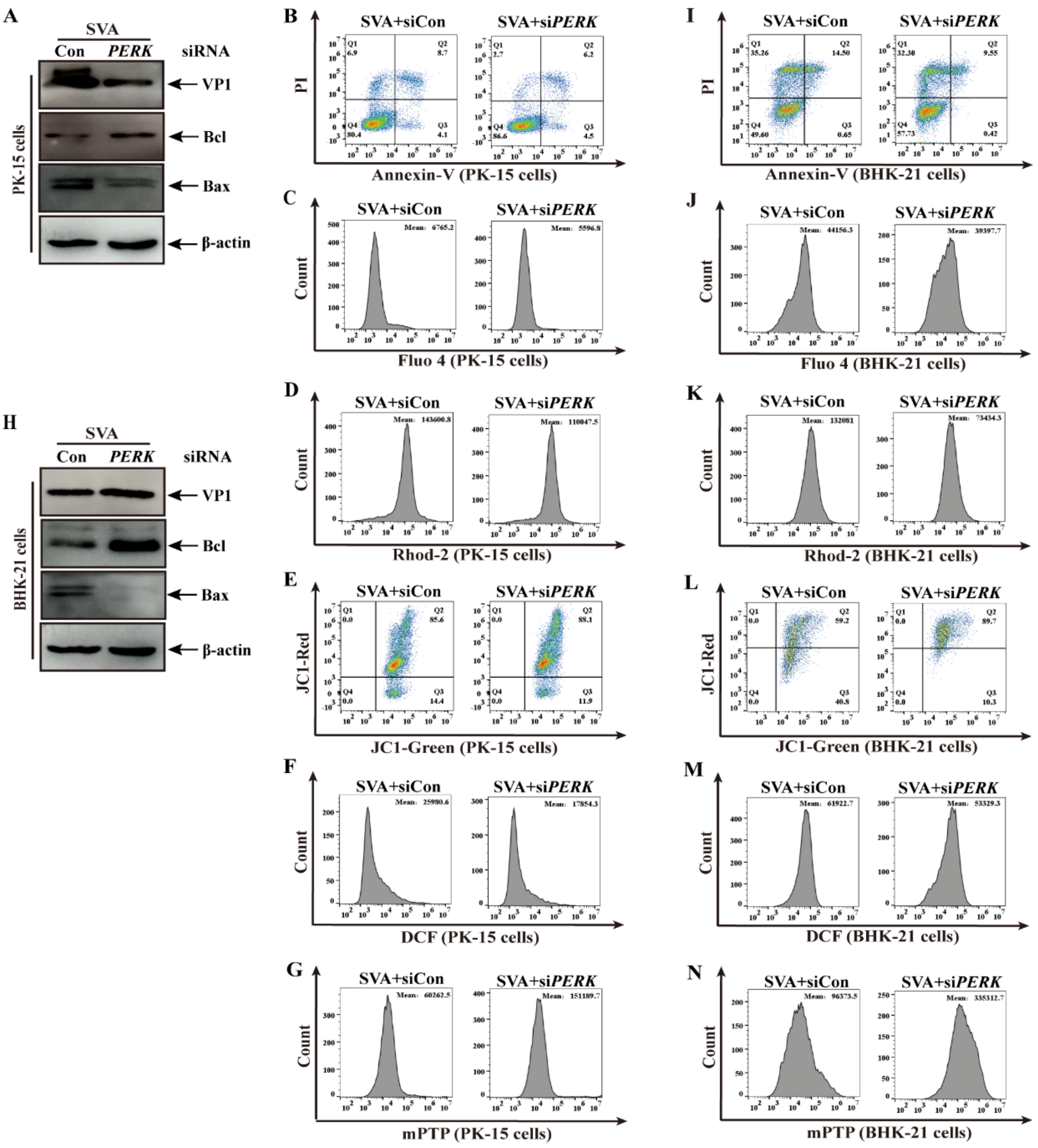

Figure S6. Flow cytometric analysis for apoptosis, cytoplasmic or mitochondrial Ca^{2+} content, MMP, mPTP, ROS in SVV-infected cells with PERK knockdown. (A and H) PK-15 (A) and BHK-21 cells (H) were infected or uninfected with SVA in the presence of siCon or si*PERK* for 24 or 9 h, followed by western blotting, as described in Fig. S1A. PK-15 or BHK-21 cells transfected with siPERK or siCon were infected with SVV and probed with different indicators, followed by detection using flow cytometry: (B and I) Apoptotic cell rates; (C and J) cytoplasmic Ca^{2+} content; (D and K) mitochondrial Ca^{2+} content; (E and L) MMP levels; (F and M) cytoplasmic ROS levels; (G and N) mPTP.
